# Multiple MYB Activators and Repressors Collaboratively Regulate the Juvenile Red Fading in Leaves of Sweetpotato

**DOI:** 10.3389/fpls.2020.00941

**Published:** 2020-06-25

**Authors:** Jiliang Deng, Danning Wu, Jie Shi, Kelly Balfour, Huafeng Wang, Guopeng Zhu, Yonghua Liu, Jian Wang, Zhixin Zhu

**Affiliations:** ^1^ Key Laboratory of Tropical Biological Resources, Ministry of Education, School of Life and Pharmaceutical Sciences, Hainan University, Haikou, China; ^2^ Department of Biology, Algoma University, Sault Sainte Marie, ON, Canada; ^3^ College of Horticulture, Hainan University, Haikou, China; ^4^ Key Laboratory of Germplasm Resources Biology of Tropical Special Ornamental Plants of Hainan Province, College of Forestry, Hainan University, Haikou, China

**Keywords:** anthocyanin, *Ipomoea*, juvenile red fading, MBW complex, MYB activators, MYB repressors, sweetpotato

## Abstract

Juvenile red fading describes the phenomenon in plants whereby red young leaves gradually turn green as they mature. While this phenomenon is commonly observed, the underlying molecular mechanism is still obscure as the classic model plants do not exhibit this process. Here, the molecular mechanism for the loss of anthocyanins during juvenile red fading were explored in the sweetpotato (*Ipomoea batatas* L.) cultivar “Chuanshan Zi”. The MYB-bHLH-WDR (MBW) regulatory complexes for anthocyanins were examined with five stages of leaf development from C1 to C5. Alternating accumulation of anthocyanins and chlorophylls caused the leaf color change. Five anthocyanin components were identified by ultra performance liquid chromatography/tandem mass spectrometry (UPLC-MS/MS), and their contents were highest at stage C2. Transcriptomic analysis showed massive gene expression alteration during leaf development. The anthocyanin structural genes expressed in sweetpotato leaves were screened and found to be highly comparable with those identified in morning glories. The screened anthocyanin regulatory genes included one bHLH (*IbbHLH2*), one WDR (*IbWDR1*), three MYB activators (*IbMYB1*, *IbMYB2,* and *IbMYB3*), and five MYB repressors (*IbMYB27*, *IbMYBx*, *IbMYB4a*, *IbMYB4b,* and *IbMYB4c*). The expression trends of MYBs were key to the red fading process: the activators were highly expressed in early red leaves and were all accompanied by simultaneously expressed MYB repressors, which may act to prevent excessive accumulation of anthocyanins. The only antagonistic repressor, *IbMYB4b*, was highly expressed in green leaves, and may be critical for declined anthocyanin content at later stages. Further functional verification of the above transcription factors were conducted by promoter activation tests. These tests showed that the MBW complexes of IbMYB1/IbMYB2/IbMYB3-IbbHLH2-IbWDR1 not only activated promoters of anthocyanin structural genes *IbCHS-D* and *IbDFR-B*, but also promoters for *IbbHLH2* and *IbMYB27*, indicating both hierarchical and feedback regulations. This study outlines the elaborate regulatory network of MBW complexes involving multiple MYBs which allow for the timely accumulation of anthocyanins in sweetpotato leaves. These results may also provide clues for similar studies of juvenile red fading in other plant species.

## Introduction

It is common in plants, especially woody plants, for newly grown leaves to be red in color and gradually fade to green as they mature (Dominy et al., 2002; Hughes et al., 2007; Gong et al., 2020). This phenomenon, termed “juvenile red fading”, depends on the alternate accumulation of anthocyanins and chlorophylls, which reflects the protective function of anthocyanins on leaves when chloroplasts are not fully developed (Hughes et al., 2007; Zhu et al., 2018). Anthocyanins belong to the flavonoids: a kind of secondary metabolite that exists widely in plants and enhances their resistance to adversity (Gould, 2004). Anthocyanins are the main red pigment of angiosperms and can accumulate in flowers and fruits to attract pollinators and fruit disseminators (Davies et al., 2012). Anthocyanins in leaves are also self-protective products for many plants, allowing them to resist a variety of biological and abiotic stress, such as strong light, diseases, pests, and herbivores (Gould, 2004; Karageorgou and Manetas, 2006). Recent investigations by Gong et al. (2020) demonstrated the importance of the juvenile red phenomenon as a chemical adaptation for the plants' defense to heavy herbivory. Although anthocyanins can be useful in plant leaves, a lot of energy is consumed during their synthesis and transport, and their reflection of red light could hinder the photosynthesis of leaves (Hughes et al., 2014). Therefore, fading of “juvenile red” is beneficial when the leaves become mature.

What is the regulatory mechanism underlying this red fading? The regulation of anthocyanin biosynthesis has been studied in great detail, revealing the MYB-bHLH-WDR (MBW) complex as the regulator for the expression of anthocyanin enzymes (Ramsay and Glover, 2005; Hichri et al., 2011). Of the three kinds of regulatory proteins, the transcription factors (TFs) belonging to the MYB family are the most crucial for the specific spatio-temporal accumulation of anthocyanins. According to recent studies, the expression of the MYBs for anthocyanins or proanthocyanins (PAs) induced the expression of their partner bHLHs to form MBW complexes (Xu et al., 2014; Zhu et al., 2015). The over-expression of anthocyanin-MYBs has produced many red-colored horticultural varieties, such as the red apple (Espley et al., 2009) and purple cauliflower (Chiu and Li, 2012). Many studies have generated high-anthocyanin plants through transgenesis with MYBs; for example, *PAP1* in Arabidopsis (Borevitz et al., 2000) and *IbMYB1* in sweetpotato (Mano et al., 2007).

However, the role played by MYBs is intriguing. To ensure appropriate amounts of anthocyanin production under diverse conditions, multiple MYBs are adopted in many plant species. In strawberry (*Fragaria* spp.), the expression of activator *FaMYB10* is accompanied by the inhibitory R2R3-type *FaMYB1* (Aharoni et al., 2001; Salvatierra et al., 2013). In petunia (*Petunia hybrida*), the R2R3-MYB inhibitor *PhMYB27* was highly expressed in shaded leaves; but under high light, *PhMYB27* was repressed while two MYB activators (*PhDPL* and *PhPHZ*) and one R3-MYB inhibitor (*PhMYBx*) were induced simultaneously (Albert et al., 2011). Elaborate regulatory network involving hierarchical and feedback regulations have been further identified among these MYB activators and repressors in studies of petunia leaves (Albert et al., 2014).

Although juvenile red fading has important biological significance, its underlying molecular mechanism remains unclear. Classic model plants, such as *Arabidopsis thaliana*, maize (*Zea mays*), rice (*Oryza sativa*), and petunia, do not exhibit this phenomenon, while many woody plants that do exhibit juvenile red fading are difficult to study. Some varieties of petunia have light-induced red foliage (Albert et al., 2011), but the red hue of young leaves associated with developmental signals was rarely reported. Using statistics from 76 woody plant species, Chen and Huang (2013) demonstrated that red young leaves have less mechanical defense than green young leaves. Hughes et al. (2007; 2008, and 2014) systematically studied the adaptive significance of juvenile red fading in multiple species, with regard to possible optical and anatomical benefits for leaves. As for the molecular mechanism behind this process, some studies have been conducted in small woody plant species. In the purple tipped foliage tea (*Camellia sinensis* L.), the MBW complex CsAN1-CsGL3/CsEGL3-CsTTG1 was attributed to the red color of young leaves, although the red fading mechanism remained obscure (Sun et al., 2016). In the tree peony (*Paeonia qiui*), the putative activator PqMYB1 and repressor PqMYB2 were identified as indicators of leaf color change, although critical experiment validations were not provided (Luo et al., 2017). Generally, the molecular mechanism behind the loss of anthocyanins during leaf development remains to be clarified.

Sweetpotato (*Ipomoea batatas* L.) is a hexaploid vine plant with 90 chromosomes (2n = 6x = 90). It is generally accepted that *I.trifida*, a diploid species from South America, is one of the two progenitors of sweetpotato, but the other ancestor has not been identified with certainty (Yang et al., 2017; Wu et al., 2018). Like many species of the genus *Ipomoea*, sweetpotato has a strong resistance to adversity, with thousands of cultivars preserved around the world. Its tuberous roots are important staple food in China, and its leaves are also highly nutritious (Wu et al., 2018). The color patterns of sweetpotato leaves appear to be independent of the roots, and many varieties display obvious juvenile red fading phenomenon. Previous studies of anthocyanins in sweetpotato have mainly focused on their purple-fleshed tuberous roots. Mano et al. (2007) isolated and identified *IbMYB1* as the gene responsible for the purple color of sweetpotato tuberous roots, and in the transgenic assays they showed that the ectopic expression of *IbMYB1* alone was sufficient for the induction of all anthocyanin structural genes. However, their RT-PCR assays did not detect expressions of *IbMYB1* nor any other anthocyanin-MYBs in red sweetpotato leaves. Dong et al. (2014) identified *IbWDR1* in the purple-fleshed roots of sweetpotato, indicating that a MBW complex was functioning in inducing the accumulation of anthocyanins. Although the regulation of anthocyanin biosynthesis was not well identified in sweetpotato, multiple studies have reported on such regulation in its close relatives *I. nil* and *I. purpurea,* which are commonly known as morning glories and grown as ornamental flowers in Asia (Morita et al., 2006; Park et al., 2007; Hoshino et al., 2016). Three anthocyanin-MYBs have been isolated from the morning glories: *InMYB1* is expressed in the flower; *InMYB2* is expressed in the petiole, stem, and roots; and the expression of *InMYB3* was not detected from any particular tissue (Morita et al., 2006). Also, the IpMYB1-IpbHLH2-IpWDR1 complex for flowers of *I. purpurea* were studied in detail in our former research (Wang et al., 2013; Zhu et al., 2015).

In this study, we explored the MBW complexes responsible for regulating the juvenile red fading phenomenon in the sweetpotato cultivar “Chuanshan Zi”. We analyzed the pigment content changes across five stages of leaf developmental (C1, C2, C3, C4, and C5), and then identified the anthocyanin components by UPLC-MS/MS. Subsequent transcriptomic analyses were conducted to explore alterations in gene expression during leaf development. The structural and regulatory genes for the anthocyanin pathway were screened by phylogeny and their expression trends were determined by qRT-PCRs. Finally, the candidate TFs for the MBW complex and representing promoters were cloned, and functional verification was conducted by promoter activation tests. Our results suggest that multiple MYB activators and repressors collaboratively participated in the process of juvenile red fading during the development of sweetpotato leaves. These results deepen our understanding of the elaborate regulatory MBW network controlling anthocyanin accumulation in plant leaves. The discovery of the MBW complexes involving multiple MYBs may provide some clues for studies of the juvenile red fading process in other plant species.

## Materials and Methods

### Plant Materials and Definition of the Leaf Developmental Stages

**Figure 1:**
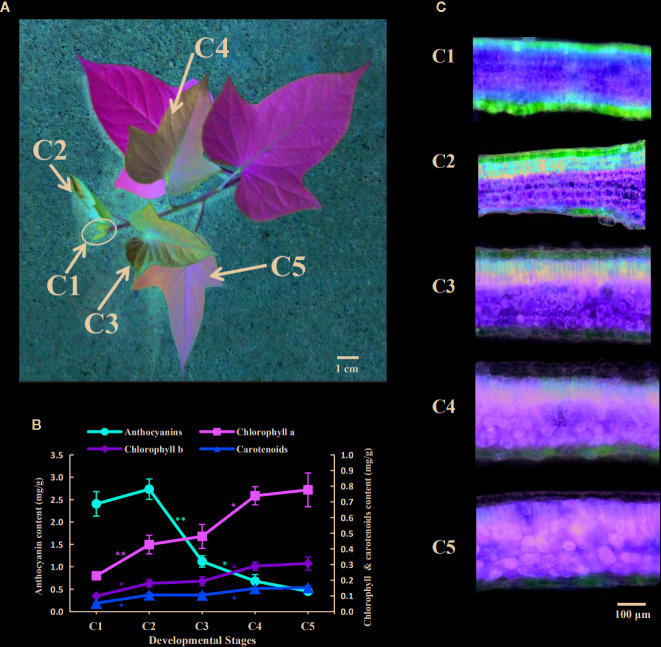
Pigment accumulation during the leaf development of the sweetpotato “Chuanshan Zi”. **(A)** The leaf development stages from C1 to C5. With the apical tip of each vine defined as the first stage C1, and the leaves beneath and along the vine are defined as stages C2 to C5. The scale bar indicates a length of 1 cm. **(B)** The pigment content alterations for the five stages, including anthocyanins, chlorophylls, and carotenoids. The bars depict the standard errors (SEs) of three biological replicates. The difference significances were tested for adjacent stages by *t*-test and asterisks indicate significant differences at p < 0.05 (*) and p < 0.01 (**). The color of the asterisks corresponds to that of the pigments. **(C)** Microscopic cross sections of leaves showing the anthocyanin distributions. Leaf sections were magnified at 200×. The scale bar indicates a length of 100 µm.

Plant materials of the sweetpotato cultivar “Chuanshan Zi” were grown in a field located on the campus of Hainan University, in the tropical coastal city of Haikou. After applying organic fertilizer to the soil, healthy vines were cut and planted in the field every three months from February to October. Plants were subjected to natural light conditions and experienced temperatures ranging from 15 to 35°C. Under such adequate nutrition and light, the color change of leaves from red to green could be clearly seen on a vine after about 1.5 to 2 months of growth. The apical tip of each vine was defined as the first development stage, C1, while the leaves further along each vine were defined as stages C2 to C5 (**Figure 1A**). Each leaf development stage would proceed to the next stage following approximately two days of growth.

Leaf samples pertaining to each of the five stages were collected in the field between the hours of 10 and 11 a.m. to avoid the influence of circadian rhythm. The collected leaves were quickly frozen in liquid nitrogen and then stored at -80°C for later usage. Leaves from three vines collected from the same stage were grounded into a fine powder with liquid nitrogen to produce one sample. Three samples were tested as biological repeats for assays including pigment measurements, UPLC-MS/MS, and qRT-PCR.

### Observation and Measurement of the Pigments

The leaves were sectioned by hand, then photographed under an optical microscope at 200× magnification. The anthocyanin contents were determined as Drumm-Herrel and Mohr (1982). Approximately 0.1 g of ground leaf sample powder was homogenized in 5 mL of 1% hydrochloric acid-methanol and extracted at 4°C in darkness for 24 h. A_530_ were determined for the supernatant. Anthocyanin content (mg/g) = 0.1×A_530_×DR mg/gFW. To determine the chlorophyll and carotenoid contents, approximately 0.1 g of ground leaf sample powder was homogenized in 10 mL of 95% ethanol. After 24 h of extraction at 4°C in darkness, the absorption values of the supernatant were measured at 665, 649, and 470 nm. Ca (mg/L) = 13.95A_665_ - 6.88A_649_, Cb (mg/L) = 24.96A_649_ - 7.32A_665_, Cx (mg/L) = (1,000A_470_ - 2.05Ca - 114.8Cb)/245. Chlorophyll a content (mg/g) = Ca×DR mg/gFW; chlorophyll b content (mg/g) = Cb×DR mg/gFW; carotenoid content (mg/g) = Cx×DR mg/gFW. In the above formulas, DR is the dilution rate and FW is the fresh weight. The data of three biological replicates were collected for the above measurements.

### Qualitative and Quantitative Analysis of Anthocyanins

UPLC-MS/MS was carried out for qualitative and quantitative measurements of anthocyanin components. For each leaf sample, 0.5 g of fine grounded powder in liquid nitrogen was homogenized in 5 mL of flavonoid extracts (methanol, MeOH) at 4°C in darkness for 24 h. Next, the obtained supernatants were further filtered through 0.22 mm reinforced nylon membranes (Shanghai ANPEL, Shanghai, China) and subjected to UPLC-MS/MS (MetWare Biotechnology Co., Ltd. Wuhan, China). Qualitative analysis was conducted by comparing the precursor ion (Q1) values, product ion (Q3) values, retention time (Rt), and fragmentation patterns of the samples with those of standards under the same conditions, which were obtained from a public database (Sigma-Aldrich, USA, http://www.sigmaaldrich.com/united-states.html), or from the self-compiled MetWare Database (MWDB, www.metware.cn). Quantitative analysis of the metabolites was based on the multiple reaction monitoring (MRM) mode, represented by chromatographic peak area integrals of the characteristic ions of each metabolite screened through the QQQ mass spectrometer. Three biological samples were tested under the same conditions for each of the five stages of leaf development.

### Transcriptomic Analysis

Total RNAs were extracted from leaf samples from stages C1 to C5 using the TRIzol method (Life technologies), and then examined by electrophoresis and NanoDrop 2000. Then, cDNA libaries were constructed with the NEBNext^®^ Ultra™ RNA Library Prep Kit for Illumina^®^ (NEB E7530, New England Biolabs). Briefly, mRNA was enriched by Oligo(dT) beads. The enriched mRNA was fragmented into short fragments with fragmentation buffer and reverse transcripted into cDNA with random primers. The second-strand cDNA was synthesized by DNA polymerase I, RNase H, dNTP, and buffer. Then the cDNA fragments were purified, end repaired, poly(A) added, and ligated to Illumina sequencing adapters. The ligation products were size selected by agarose gel electrophoresis, PCR amplified, and sequenced using Illumina HiSeq™4000 by Gene Denovo Biotechnology Co. (Guangzhou, China). High quality clean reads were first aligned to de-novo cognate assemblies of “Chuanshan Zi” and then mapped to the reference genome by TopHat2 after the release of the genomic sequence of the sweetpotato cultivar “Taizhong 6” (Yang et al., 2017). No fundamental differences were displayed in the comprehensive analysis for the categories of expressed genes; however, subsequent analysis with the reference genome for specific gene sequences showed considerable incompleteness and inaccuracy that hindered further investigation. To bypass these issues, the alignment to the de-novo cognate assemblies was used as the main data, while the alignment to the “Taizhong 6” reference genome was used as auxiliary data for subsequent homologous cloning of the promoters, together with genomes of *I. nil* (Hoshino et al., 2016), *I. trifida*, and *I. triloba* (Wu et al., 2018). The RNA-Seq data are available from the NCBI Short Read Archive No. PRJNA612413 (http://www.ncbi.nlm.nih.gov/).

### Screening of the Structural Genes of the Anthocyanin Pathway

As thousands of genes were differentially expressed for adjacent developmental stages, screens of anthocyanin structural genes were conducted mainly on the basis of KEGG pathway annotation. Genes assorted to the pathway from ko00941–ko00944 were searched for the anthocyanin structural genes, which scattered in the multiple flavonoid branches. Unigenes that were too short in length were excluded. The candidate genes were then blasted in NCBI for further verification. Most of the screened genes were named according to the *I. nil* or *I. purpurea* homologues. The pathway genes for chlorophyll and carotenoid metabolism were also screened to reveal the expression trends of their biosynthetic processes. The carotenoid biosynthetic genes were mainly screened from ko00900 and ko01062 based on KEGG annotation. The chlorophyll biosynthetic genes were mainly screened by local blast using genes from Arabidopsis as queries.

### Phylogenetic Analysis for the Anthocyanin MYB, bHLH, and WDR Genes

Phylogenetic analysis using protein sequences was applied to search for the anthocyanin/flavonoid-related *MYB*, *bHLH,* and *WDR* genes. The amino acid sequences of 62 R2R3-MYBs and 112 bHLHs were extracted from the transcriptomic data. The MYBs and bHLHs from Arabidopsis and other model species were used to assist the search (**Supplementary Table S1**). Due to large differences of the C-terminal sequences of MYB proteins, even among functionally equivalent members (Dubos et al., 2010), only the R2R3 domains were used in the MYB phylogenetic analysis. The R2R3-MYB subfamilies were classified according to Dubos et al. (2010). The bHLH members clustered within SGIIIf were regarded as flavonoid-related candidates according to Pires and Dolan (2010). The flavonoid-related WDRs are very conserved in plants and were directly obtained by local blast using *AtTTG1* as the query. The candidate R3-MYBs were obtained by local blast using *AtCPC*, *AtTRY*, *AtETC1,* and *AtETC2* as queries and were further confirmed by blast in NCBI. Phylogenetic trees were constructed by MEGA X (Kumar et al., 2018). Parameters for the NJ tree were set as p-distance model and pairwise deletion with the bootstrap value as 1,000. All the screened anthocyanin-genes were blased for the location/ID in the published genome of *I. batatas* cultivar “Taizhong 6” (Yang et al., 2017) with the criteria of E-value < 1×10^-5^ (**Supplementary Table S2**).

### qRT-PCRs

RNA samples were extracted by Eastep^®^ Super Total RNA Extraction Kit (Promega) from three biological samples for each of the five stages of leaf development. After quality verification by electrophoresis and NanoDrop 2000, the RNAs were reverse transcribed by Eastep^®^ RT Master Mix Kit (Promega) and quantified with specific primers following the instruction of Eastep^®^ qPCR Master Mix Kit (Promega) (primers are listed in **Supplementary Table S3**). Relative mRNA levels were calculated by comparative quantification (Pfaffl, 2001) to the geometric mean of the two housekeeping genes PURA1 and ARF.

### Cloning of TFs and Promoters

The candidate members for MBW complexes were cloned from the cDNA of the “Chuanshan Zi” leaves. Most of these TFs were successfully cloned with primers designed from assembled unigene sequences, indicating the reliability of the self-aligned de-novo reference database. Primers for promoter cloning were designed by the available conspecific sequences from released genomes of *I. batatas*, *I. trifida,* and *I. triloba* (Yang et al., 2017; Wu et al., 2018). Standard PCRs were applied to the leaf genomic DNA and six promoters were successfully cloned, including promoters for *IbCHS-D*, *IbCHS-E*, *IbDFR-B*, *IbbHLH2*, *IbMYB27,* and *IbMYB4c*. Pfu DNA Polymerase (Sangon Biotech) was used for the cloning, and at least four clones were sequenced. Further experiments were carried out with the sequences represented by more than two clones. Sequence data for the cloned TFs and promoters can be found in NCBI under the accession numbers MT231489 to MT231504. The coding sequences for most of the screened structural genes were also cloned as above, and can be found under the accession numbers MT557573 to MT557584 (**Supplementary Table S2**).

### Promoter Activation Tests by Dual Luciferase Assays

The responsiveness of the promoters to the MBW complex was tested by dual luciferase assays, with firefly luciferase (LUC) as the reporter and renilla luciferase (RUC) as the reference. Cloned TFs and promoters were integrated into adapted pJIT163 vectors as reported by Wang et al. (2013). The complete coding regions of the TFs were integrated into pJIT163 to construct the *35S::TF* effector vectors. The promoters were ligated into pJIT163-LUC to replace the *CaMV35S* resulting *promoter::LUC* reporter vectors. The vector pJIT163-RUC was used as a reference.

Sequence verified vectors were introduced into the young leaves of the *I. nil wdr1* mutants by particle bombardment (GJ-1000 High-pressure Gene Gun). A construct mixture (0.4 μg effector, 0.4 μg reporter, and 0.1 μg reference for each shot) was coated with 50 mg/mL gold microparticles (Bio-Rad) at a ratio of 2.0 μL per 1.0 μg of plasmid DNA. The complex was then mixed with 2.5 M CaCl_2_ and 0.1 M spermidine in a ratio of 1:5:2. A pressure of 4.5 MPa and a distance of 6 cm were used for the bombardment. The bombarded leaves were kept in the dark at 25°C for about 30 h. The luciferase activities were detected by Glomax 20/20 luminometer following the manufacturer's instruction of the Dual-Luciferase^®^ Reporter (DLR™) Assay System (Promega). The mean values and SEs were obtained from three biological replicates for each of the five stages of leaf development.

## Results

### Pigment Alteration During Leaf Development

From the stages C1 to C5, the color of the “Chuanshan Zi” leaves gradually changed from red to green, with obvious greening occurring at the stage C4 (**Figure 1A**). The pigment contents were measured, including anthocyanins, chlorophylls, and carotenoids (**Figure 1B**). The contents of red anthocyanins reached maximum in C2 and decreased significantly in the stages thereafter, whereas contents of the green chlorophylls and the orange carotenoids, which are photosynthesis-related pigments, gradually increased during leaf development.

The pigment distributions were shown by cross sections of the leaves (**Figure 1C**). In stage C1, the mesophyll cells were not yet differentiated, and the red anthocyanins accumulated in the upper and lower epidermis and adjacent mesophyll. In stages C2 to C4, the mesophyll gradually differentiated into palisade and spongy tissues and the anthocyanins were distributed in the epidermis and the upper palisade tissues. The epidermal cells appeared bright red due to the lack of chlorophyll, while the palisade cells containing anthocyanin appeared brown due to the mixing color of anthocyanins and chlorophylls. With the progressed growth of leaves, the anthocyanins in the epidermis and palisade tissues gradually faded. By the C5 stage, only a slight red color was observed in the lower epidermis.

### Qualitative and Quantitative Analysis of Anthocyanins

**Figure 2:**
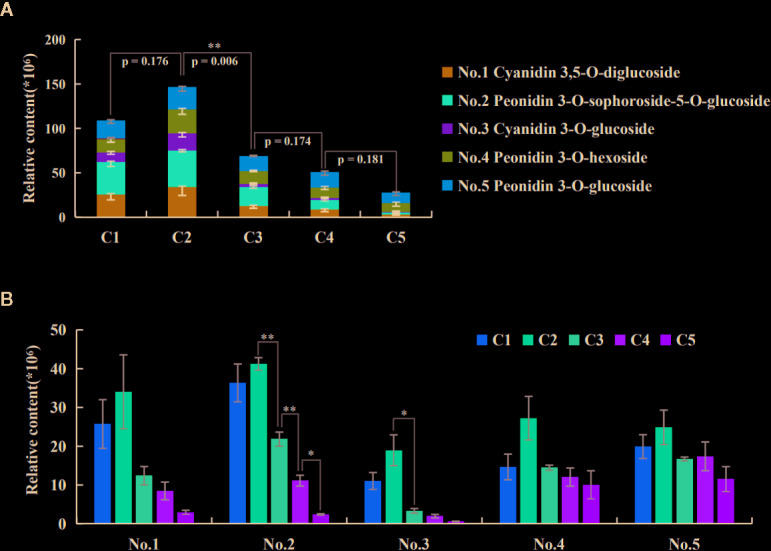
Quantities of the five anthocyanin components during leaf development. **(A)** The trend of total anthocyanin content. The p-value by *t*-test for adjacent stages were displayed, with extremely significant results only for the C2-vs-C3 comparison. The negative SE bars of three biological replicates were shown for each component. **(B)** Trends of each anthocyanin component along the five stages. The bars indicate the SEs of three biological replicates. The difference significances were tested for adjacent stages by *t*-test and asterisks indicate significant differences at p < 0.05 (*) and p < 0.01 (**).

A total of five anthocyanins were characterized by UPLC-MS/MS, including two cyanidin- and three peonidin-derivatives (**Table 1**). The quantitative analysis showed the trend of total anthocyanin content (**Figure 2A**) being consistent with previous optical measurement (**Figure 1B**), and only the C2-vs-C3 comparison was extremely significant (p=0.005) for adjacent stages. The relative quantities of all five anthocyanin components reached a peak at C2 (**Figure 2B**). For comparisons of adjacent stages, no significance was displayed for cyanidin 3,5-*O*-diglucoside (No. 1), peonidin 3-*O*-hexoside (No. 4), and peonidin 3-*O*-glucoside (No. 5). The significant difference for total anthocyanin in C2-vs-C3 was mainly attributed to peonidin 3-*O*-sophoroside-5-*O*-glucoside (No. 2) and cyanidin 3-*O*-glucoside (No. 3). The components of No. 1, 2, and 3 decreased to very low levels in the later stages of leaf development, while no significant decreases occurred for No. 4 and 5 during these stages. This indicates that anthocyanins located within the lower epidermis at the C5 stage are likely the components of No. 4 and 5.

**Table 1:** A list of the five anthocyanin components identified in sweetpotato leaves.

No.	Anthocyanins	Ionization model	Rt (min)	Precursor ions (Q1) (Da)	Product ions (Q3) (Da)	Molecular Weight (Da)
1	Cyanidin 3,5-*O*-diglucoside	Protonated	2.08	611.00	287.00	611.00
2	Peonidin 3-*O*-sophoroside-5-*O*-glucoside	Protonated	2.23	787.23	463.40	787.23
3	Cyanidin 3-*O*-glucoside	Protonated	2.59	449.10	287.30	449.10
4	Peonidin 3-*O*-hexoside	Protonated	2.89	463.10	301.00	463.12
5	Peonidin 3-*O*-glucoside	[M-Cl]^+^	2.94	463.10	301.10	498.09

Among these five anthocyanin components, No. 1–4 were detected in the ionization model of “protonated” and arranged by the retention time (Rt), while No.5 was detected in the ionization model of “[M-Cl]^+^”.

### Comprehensive Transcriptomic Analysis for the Five Leaf Stages

**Figure 3:**
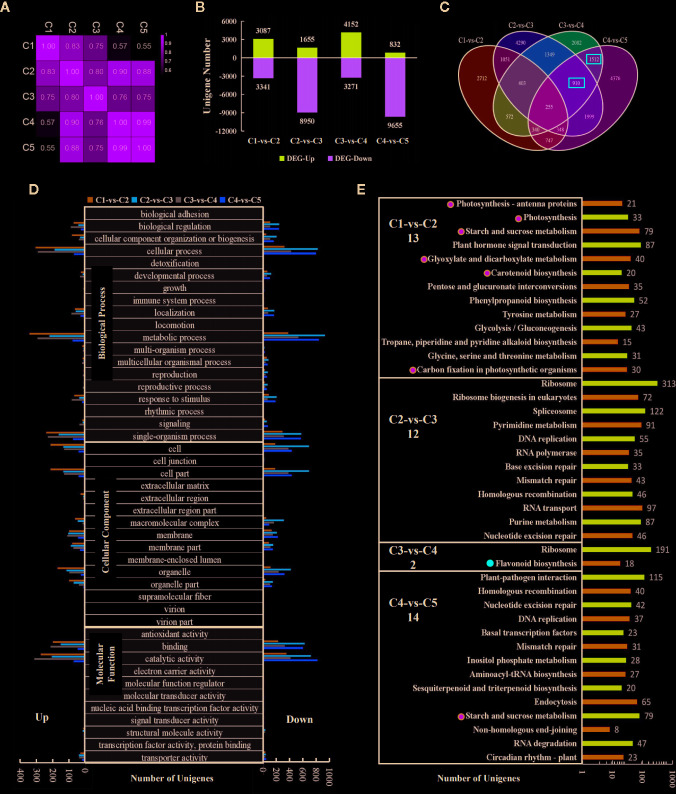
Transcriptomic analysis for the five stages of leaf development in “Chuanshan Zi”. **(A)** The Pearson's coefficients for all expressed genes. **(B)** The differentially expressed gene (DEG) numbers for adjacent leaf stages, screened with the criteria of |log_2_FC| > 1 and FDR < 0.05. **(C)** The Venn diagram analysis of the DEGs. The red boxes indicate the main DEG groups for flavonoid/anthocyanin unigenes. **(D)** The gene ontology (GO) functional categorization of the DEGs. The left and right panels showed the up- and down-regulated genes, respectively. **(E)** The KEGG enrichment analysis for metabolism pathways. The items related to photosynthesis were emphasized with green dots, and those related to the flavonoid/anthocyanin biosynthesis pathway with red dots. Only pathways with significant difference were displayed. The number of the significant pathways were shown under the names of comparison groups.

To screen for anthocyanin-related genes in sweetpotato leaves, RNA-seq were applied to leaves of “Chuanshan Zi”. A total of 50,554 unigenes were obtained with an average length of 931 nt. The Pearson's correlation coefficients for all expressed genes are provided (**Figure 3A**). C1 displayed a gradually decreasing correlation with the subsequent four stages. The correlation between C4 and C5 reached 0.99, while C3 showed relatively poor correlation with the other four stages.

With the criteria of |log_2_FC| > 1 and FDR < 0.05, the differentially expressed genes (DEGs) between adjacent leaf stages were screened (**Figures 3B–E**). Thousands of DEGs were detected in each of the comparisons, even for the highly correlated C4 and C5, reflecting vigorous cell activities during leaf development (**Figure 3B**). Both C2-vs-C3 and C4-vs-C5 involved massive down-regulated genes. From C2 to C3, the leaves fully extended and began to fade, accompanied by the decreased expression of 8,950 genes. From C4 to C5, the leaves reached the state of maturity and 9,655 genes were down-regulated. The total number of expressed genes decreased from 49,614 in C1 to 43,566 in C5.

The Venn diagram analysis (**Figure 3C**) showed that the majority of the DEGs were unique within each comparison. Flavonoid/anthocyanin-related unigenes mainly enriched in the 1,512 (C3-vs-C4 and C4-vs-C5) and 910 (C2-vs-C3, C3-vs-C4, and C4-vs-C5) groups (indicated by red boxes). The gene ontology (GO) functional categorization of the DEGs are shown in **Figure 3D**. For biological processes, the DEGs enriched mostly in “cellular process”, “metallic process”, and “single-organism process”. For cellular components, the DEGs enriched mostly in “cell” and “cell part”, followed by “organelle”, “organelle part”, “membrane”, and “membrane part”. For molecular functions, the majority of DEGs enriched in “catalytic activity” and “binding”. The above results are consistent with the occurrence of vigorous cell development in the leaves.

KEGG enrichment was conducted for pathway analysis. The pathways with both significant p-values and q-values were exhibited (**Figure 3E**). In the C1-vs-C2 group, 13 metabolic pathways were enriched, six of which were directly related to photosynthesis (indicated by green dots). In the C2-vs-C3 group, the genomic DNA seemed to have undergone massive changes to adapt to the great gene expression alteration. Although the color change phenotype is obvious for the leaves, the flavonoid/anthocyanin pathways enriched significantly only in the C3-vs-C4 group (indicated by the red dot), including structural genes *CHS*, *CHI*, *F3H*, *F3'H*, *DFR*, and *ANS*. Further examination of the top 20 enriched pathways showed that the flavonoid pathway ranked 16^th^ in the C4-vs-C5 group (with 14 significant pathways) and was very close to the threshold of significance.

### Identification of the Structural Genes of the Anthocyanin Pathway

Through KEGG annotation, the structural genes of the anthocyanin pathway were obtained (**Table 2**). Except for *CHS*, *DFR,* and *F3'5'H*, all of the other structural genes had only one single copy expressed in leaves. Two copies of *CHS* were found, corresponding to *CHS-D* and *CHS-E,* which were responsible for the coloring of flower limb and tube in morning glories, respectively (Durbin et al., 2001). No *F3'5'H* was detected, which was consistent with the loss of *F3'5'H* in the genus *Ipomoea* (Zufall and Rausher, 2004). Three copies of *DFR* were screened out, corresponding to *DFR-A*, *DFR-B,* and *DFR-C* of morning glories. The study of *I. purpurea* showed that although the three *DFR* copies are paralogous, only *DFR-B* is involved in anthocyanin synthesis, while the functions of the other two copies are unknown (Des Marais and Rausher, 2008). Except for *IbCHS-E* and *Ib5GT2*, the other structural genes could be found in the C3-vs-C4 DEG group, and most of them were also found in C4-vs-C5 DEG group. The key structural gene *Ib3GGT1* also appeared in the DEG group of C2-vs-C3.

**Table 2:** Candidate structural and regulatory genes for anthocyanin biosynthesis in sweetpotato.

Candidate genes	No.	Unigenes	Homologous genes	Names	DEG group
*CHS*	2	Unigene0037527	*IpCHS-D/InCHS-D*	*IbCHS-D*	C3-vs-C4, C4-vs-C5
		Unigene0004388	*IpCHS-E/InCHS-E*	*IbCHS-E*	none
*CHI*	1	Unigene0039450	*IpCHI/InCHI*	*IbCHI*	C3-vs-C4, C4-vs-C5
*F3H*	1	Unigene0032882	*IpF3H/InF3H*	*IbF3H*	C3-vs-C4, C4-vs-C5
*F3'H*	1	Unigene0005410	*IpF3'H/InF3'H*	*IbF3'H*	C3-vs-C4, C4-vs-C5
*F3'5'H*	0				
*DFR*	3	Unigene0004509	*IpDFR-A/InDFR-A*	*IbDFR-A^#^*	C3-vs-C4, C4-vs-C5
		Unigene0009726	*IpDFR-B/InDFR-B*	*IbDFR-B*	C3-vs-C4, C4-vs-C5
		Unigene0012538	*IpDFR-C/InDFR-C*	IbDFR-C *^,#^	C2-vs-C3, C3-vs-C4, C4-vs-C5
*ANS*	1	Unigene0006788	*IpANS/InANS*	*IbANS*	C3-vs-C4, C4-vs-C5
*3GT*	1	Unigene0008671	*Ip3GT/In3GT*	*Ib3GT*	C3-vs-C4, C4-vs-C5
*5GT*	2	Unigene0037564	*Ip5GT/In5GT*	*Ib5GT1*	C1-vs-C2, C3-vs-C4
		Unigene0036946	*Ip5GT/In5GT*	*Ib5GT2*	none
*3GGT*		Unigene0011484	*Ip3GGT/In3GGT*	*Ib3GGT1*	C2-vs-C3, C3-vs-C4, C4-vs-C5
R2R3-MYB activators	3	Unigene0031238	*IbMYB1/IpMYB1/InMYB1*	*IbMYB1*	C2-vs-C3, C3-vs-C4, C4-vs-C5
		Unigene0031237	*InMYB2/InMYB3/IbMYB1*	*IbMYB2*	none
		Unigene0023598	*InMYB3/InMYB2/IbMYB1*	*IbMYB3*	C2-vs-C3, C3-vs-C4, C4-vs-C5
R2R3-MYB repressors	4	Unigene0001224	*PhMYB4/AtMYB4*	*IbMYB4a*	C2-vs-C3, C3-vs-C4
		Unigene0003763	*AtMYB4*	*IbMYB4b*	none
		Unigene0021216	*AtMYB4*	*IbMYB4c*	none
		Unigene0009722	*PhMYB27/FaMYB1*	*IbMYB27*	C3-vs-C4, C4-vs-C5
R3-MYB repressors	1	Unigene0001816	*PhMYBx*	*IbMYBx*	C2-vs-C3
bHLH	3	Unigene0010710	*InbHLH1*	*IbbHLH1* ^*^	C3-vs-C4, C4-vs-C5
		Unigene0041364	*InbHLH2/IpbHLH2*	*IbbHLH2*	C2-vs-C3, C3-vs-C4, C4-vs-C5
		Unigene0018919	*InbHLH3*	*IbbHLH3* ^*^	C2-vs-C3
WDR	2	Unigene0025722	*InWDR1/IpWDR1/ZmPAC1*	*IbWDR1*	none
		Unigene0012406	*InWDR2/IpWDR2/ZmMP1*	*IbWDR2^#^*	none

^#^indicate the genes that are highly homologous to the anthocyanin genes but have functions unrelated to anthocyanin synthesis. ^*^ indicate the genes with extremely low expression levels, as revealed by RPKMs and qRT-PCR.

CHS, chalcone synthase; CHI, chalcone isomerase; F3H, flavanone 3-dioxygenase; F3'H, flavanoid 3'-hydroxylase; F3'5'H, Flavonoid-3'5'-hydroxylase; DFR, dihydroflavonol-4-reductase; ANS, anthocyanidin synthase; 3GT, anthocyanidin 3-O-glcosyl-transferase; 5GT, anthocyanidin 5-O-glcosyl-transferase; 3GGT, anthocyanidin 3-O-glucoside-2''-O-glucosyltransferase.

### Screen of the Anthocyanin-Related MYB, bHLH, and WDR Genes

**Figure 4:**
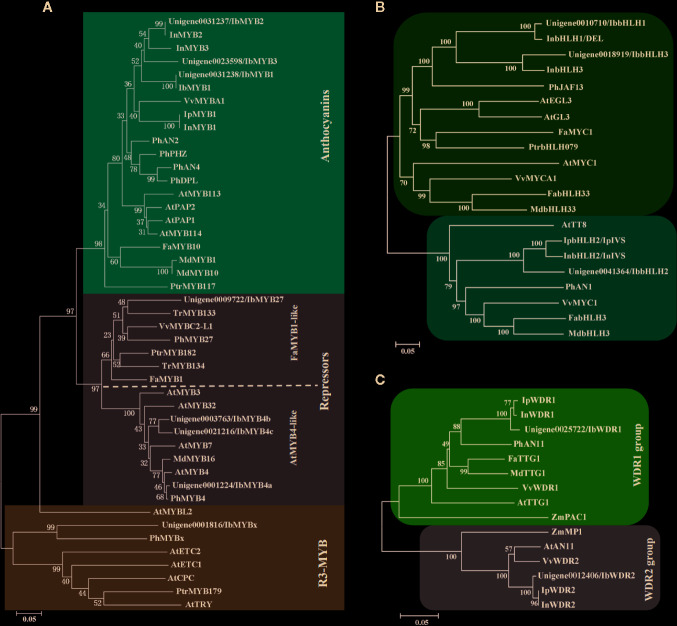
Phylogenetic analysis of the putative flavonoid/anthocyanin-related MYB, bHLH, and WDR members. **(A)** Phylogenetic relationship of putative MYBs in sweetpotato and function verified anthocyanin-related MYBs in other species. The red and grey shaded areas indicate the R2R3-MYB activators and repressors, respectively. The blue shaded area shows the R3-MYB repressors. The R2R3-MYB repressors were further classified as AtMYB4-like and FaMYB1-like groups. **(B)** Phylogenetic relationship of flavonoid-related bHLHs. **(C)** Phylogenetic relationship of WDRs from groups of WDR1 and WDR2. Only the WDR1 group members are functional in MBW complexes.

The 62 R2R3-MYBs extracted from the transcriptome were first aligned with the well-classified 126 Arabidopsis R2R3-MYBs (**Supplementary Figure S1**), and then aligned with functional verified flavonoid-related MYBs in other species (**Supplementary Figure S2**). The phylogenetic relationship of the screened MYBs with other classic anthocyanin-related MYBs was outlined (**Figure 4A**, **Supplementary Table S1**). The flavonoid-related R2R3-MYBs mainly clustered in four subfamilies (SGs): SG4 (repressors), SG5 (PAs), SG6 (anthocyanins), and SG7 (flavonols) (Dubos et al., 2010). Three unigenes clustered in the SG6 subfamily (unigene0031238/*IbMYB1*, unigene0031237/*IbMYB2*, and unigene0023598/*IbMYB3*) were named after their homologous genes *InMYB1*, *InMYB2*, and *InMYB3* from *I. nil* (**Figure 4A**, **Table 2**). Unigene0001224/*IbMYB4a*, unigene0003763/*IbMYB4b*, and unigene0021216/*IbMYB4c* clustered as AtMYB4-like SG4 members, with unigene0009722/*IbMYB27* as a FaMYB1-like member and unigene0001816/*IbMYBx* as a PhMYBx-like R3-MYB (**Figure 4A**, **Table 2**).

The plant bHLH family participates in many biological processes, among which only members from the SGIIIf subfamily contribute to flavonoid synthesis (Pires and Dolan, 2010). The 112 sweetpotato bHLHs extracted were first aligned with the Arabidopsis bHLHs (**Supplementary Figure S3**) and three unigenes in the SGIIIf subfamily (unigene0010710, unigene0041364, and unigene0018919) were found. Further phylogenetic analysis conducted with anthocyanin-related bHLHs (**Figure 4B**, **Table 2**) showed that these three unigenes were homologous with *InbHLH1*, *InbHLH2*, and *InbHLH3* in *I. nil* (Morita et al., 2006).

The WDRs of the MBW complexes are highly conserved, with only a single copy, generally called *WDR1*, being found in most plants. Another copy, *WDR2*, with a similar sequence but an unidentified function had been cloned in many species: *ZmMP1*, *AtAN11,* and *VvWDR2* (Carey et al., 2004; Morita et al., 2006; Matus et al., 2010). A recent study of AtAN11 (also known as Light-regulated WD1, or LWD1) suggested that it functions as a clock protein, contributing to the regulation of circadian period length and photoperiodic flowering (Wu et al., 2016). We used *AtTTG1* as the query and got unigene0025722 and unigene0012406, corresponding to *InWDR1* and *InWDR2*, respectively (**Figure 4C**). Only unigene0025722/*IbWDR1* was used in the following analysis. Most of the screened TFs could be found in DEG groups of C2-vs-C3, C3-vs-C4, and C4-vs-C5 (**Table 2**), which was consistent with the structural genes.

All the screened anthocyanin-genes were blased for the location/ID in the published genome of *I. batatas* cultivar “Taizhong 6” (Yang et al., 2017) with the criteria of E-value < 1×10^-5^ (**Supplementary Table S2**). Most of the unigenes could be assigned to multiple locations, possibly as a result of the hexaploid state of the genome. Three of the confirmed genes (*IbMYB3*, *IbWDR1*, and *IbWDR2*) could not be assigned to any locations in the reference genome of the “Taizhong 6”.

### Function Prediction from Sequence Alignment for Screened MYBs

**Figure 5:**
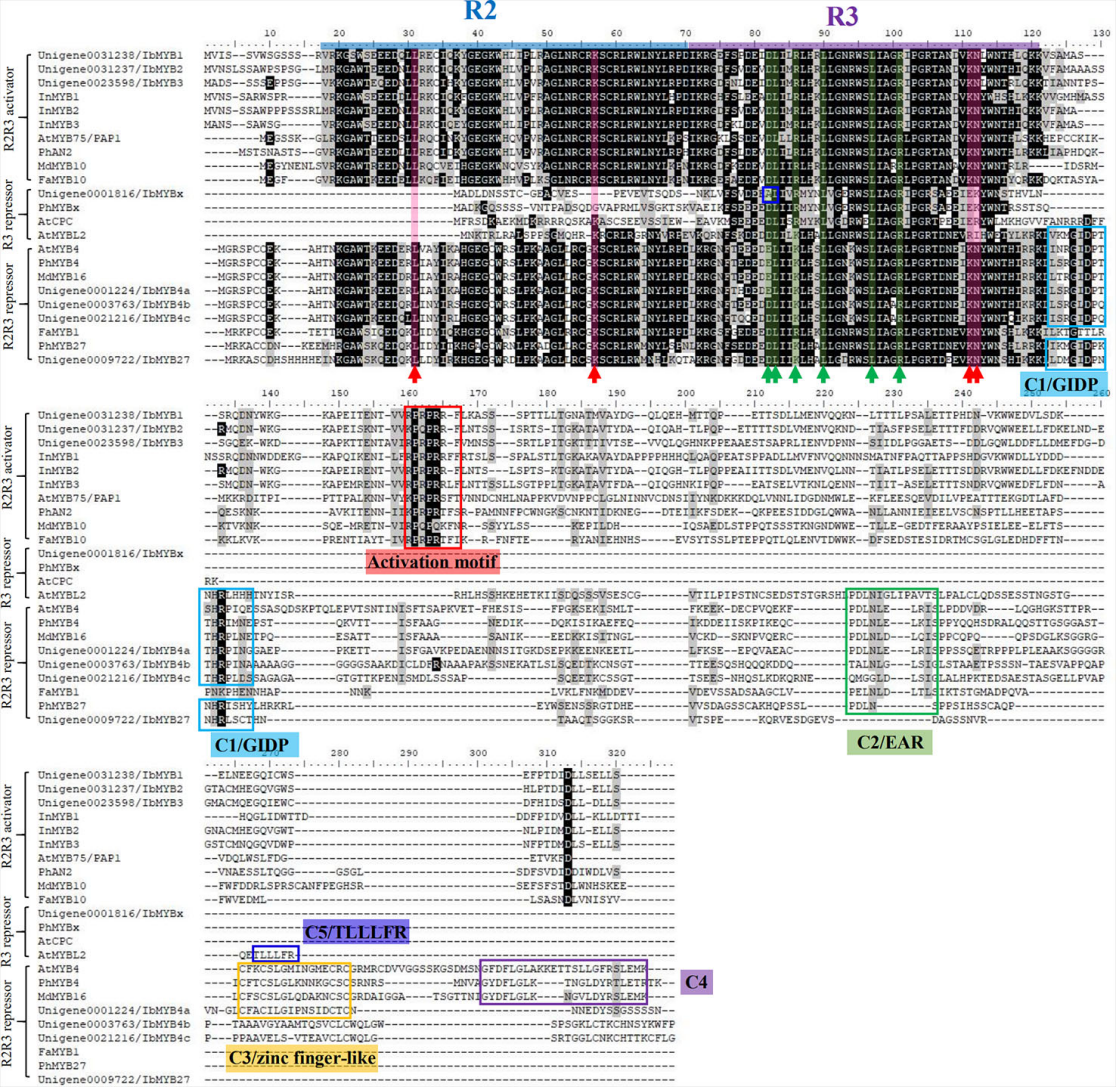
Protein sequence alignment for the putative MYBs with function verified homologous genes of other plant species. The blue and purple lines represent the R2 and R3 domains, respectively. The red arrow with the red shadow indicates the key sites for MYBs' binding to the DNA (Ording et al., 1994). The green arrow with the green shadow indicates the ^D^/_E_Lx_2_^R^/_K_x_3_Lx_6_Lx_3_R motif for MYBs' binding to their bHLH partners (Zimmermann et al., 2004). The blue box in the R3 region of IbMYBx outlines the mutation that might interfere with its binding to bHLHs. The red box indicates the characteristic KPRPR^S^/_T_F activation motif of anthocyanin MYBs in their C-terminus, as summarized by Stracke et al. (2010). The other colored boxes of at the C-terminus region indicate the characteristic repressive motifs in MYB repressors, including motifs of C1/GIDP, C2/EAR, C3/zinc finger-like, C4, and C5/TLLLFR (Cavallini et al., 2015).

The multiple MYBs screened by phylogeny were cloned and aligned with classic anthocyanin-MYBs in model plants like Arabidopsis, petunia, apple, strawberry*, I. purpurea*, and *I. nil* to predict their possible functions (**Figure 5**, **Table 3**).

**Table 3:** Predicted functions for the candidate MYB activators and repressors from the protein sequence alignments.

Predicted functions	IbMYB1	IbMYB2	IbMYB3	IbMYB27	IbMYBx	IbMYB4a	IbMYB4b	IbMYB4c
DNA binding	Yes	Yes	Yes	Yes	No	Yes	Yes	Yes
bHLH binding	Yes	Yes	Yes	Yes	Yes*	Yes	Yes	Yes
Transcription activation	Yes	Yes	Yes	No	No	No	No	No
Passive repression by binding DNA	No	No	No	Yes	No	Yes	Yes	Yes
Passive repression by binding bHLH	No	No	No	Yes	Yes*	Yes	Yes	Yes
Active repression by repressive motifs	No	No	No	Yes	No	Yes	Yes	Yes

* indicate that the function prediction is uncertain.

R2R3-MYBs have highly homologous R2R3 domains, while the R3-MYBs only possess the homologous domain of R3. The four critical DNA-binding amino acids for the R2R3-MYB family, resolved by Ording et al. (1994), were conserved among the R2R3-MYBs (**Figure 5**, indicated by red arrows and red shadow) but were lost or mutated for the R3-MYBs. This situation is consistent with the fact that R3-MYBs do not bind to DNA, and we predict that IbMYBx is also unable to bind DNA (**Table 3**).

The bHLH recognition motif (^D^/_E_Lx_2_^R^/_K_x_3_Lx_6_Lx_3_R) for the binding of MYBs to their bHLH partners were within the R3 domain (**Figure 5**, indicated by green arrows and green shadow) and were conserved among the aligned MYBs, except for IbMYBx. The assumed MYB activators displayed the conserved sequence of KPRPR^S^/_T_F in their C-terminus (**Figure 5**, indicated by red box), which was the characteristic motif of the anthocyanin-MYB activators as summarized by Stracke et al. (2010). It is speculated that IbMYB1, IbMYB2, and IbMYB3 could all activate anthocyanin synthesis in sweetpotato (**Table 3**).

Cao et al. (2017) summarized the inhibitory MYBs of the flavonoid metabolism as three groups—CPC/TRY-like (R3-MYB), MYB4-like (R2R3-MYB), and FaMYB1-like (R2R3-MYB)—which was consistent with our results of phylogeny (**Figure 4A**). The inhibitory function of R3-MYBs was mainly implemented by passive repression of the competitive binding of bHLHs. For flavonoid-related R2R3-MYB repressors, the inhibitory mechanism varies (**Table 3**). The characteristic inhibitory domains include five motifs: C1/GIDP (lsrGIDPx^T^/_N_HR), C2/EAR (pdLNL^D^/_E_Lxi^G^/_S_), C3/zinc finger-like, C4 (dFLGL and LD^F^/_Y_RxLEMK), and C5/TLLLFR (Stracke et al., 2010; Cavallini et al., 2015; Schwinn et al., 2016; Ma et al., 2018; Ma and Constabel, 2019). The protein sequence alignment determined that IbMYB4a had three repression motifs (C1, C2, and C3), IbMYB4b and IbMYB4c each displayed two repression motifs (C1 and C2), and IbMYB27 only displayed the C1 motif (**Figure 5**). The C5/TLLLFR motif was present only in AtMYBL2 among the chosen MYBs. AtMYBL2 was reported as R3-MYB, but the alignment indicated that it is most likely a truncated R2R3-MYB repressor—it lost the former half of the R2 region but retains a complete C-terminal that contains the C1, C2, and C5 motifs. In contrast, IbMYBx is a typical R3-MYB protein with very short C-terminals. The predicted mechanisms of repression for these MYBs were summarized in **Table 3**.

### Expressions of the Structural and Regulatory Genes of the Anthocyanin Pathway

**Figure 6:**
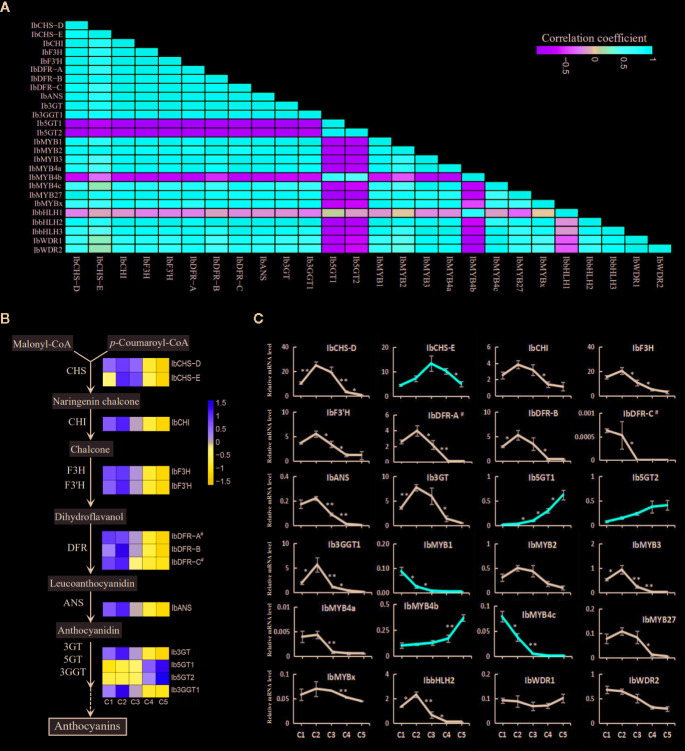
The expression of the structural and regulatory genes of the anthocyanin pathway along the five leaf stages. **(A)** The Pearson's coefficients of the screened structural and regulatory genes based on the RPKMs. **(B)** The expression level of the structural genes along the anthocyanin pathway. Heatmaps were constructed based on the values of log_2_RPKM. **(C)** The relative expression level assayed by qRT-PCR for the putative structural genes and regulatory MYB, bHLH, and WDR members. Most of the genes showed an expression trend consistent with the total anthocyanin content. Genes with other trends are represented by red lines. The X-axes show the five developmental stages and the Y-axes show the relative mRNA levels, with the error bars based on three biological replicates. The difference significances were tested for adjacent stages by *t*-test and asterisks indicate significant differences at p < 0.05 (*) and p < 0.01 (**).

Pearson's correlation coefficients were calculated from the RPKMs of the screened genes (**Figure 6A**). All the structural genes, except for *IbCHS-E*, *Ib5GT1,* and *Ib5GT2,* showed significant positive correlations with the three MYB activators. The *IbCHS-E* was significantly correlated only with *IbCHS-D* and *IbMYB2*. The *Ib5GT1* and *Ib5GT2* were negatively correlated with most of the other genes. For the regulatory genes, *IbMYB4b* was negatively correlated with most of the other genes. The *IbbHLH1*, *IbWDR1,* and *IbWDR2* were not significantly correlated with other genes.

The expression trends of the regulatory and structural genes of the anthocyanin pathway based on the RPKM values (**Figure 6B**) were generally consistent with the results of qRT-PCR (**Figure 6C**). However, more regulatory and structural genes were shown to drop in their expression between C2 and C3 stages by qRT-PCR than by RNA-seq analyses (**Figure 6C**, **Table 2**), including the TFs *IbMYB1, IbMYB3, IbMYB4a, IbMYB4c,* and *IbbHLH2*, and the structural genes *IbF3H, IbF3'H, IbANS,* and *Ib3GGT1.* Among the examined genes, *IbbHLH1* and *IbbHLH3* displayed high Ct values in qRT-PCR, indicating extremely low expression levels, and were not included in **Figure 6C**. Three expression trends emerged for the rest of the genes. The first major trend, which peaked in stage C2 and then decreased, included the majority of the genes and was consistent with the overall trend of anthocyanin accumulation (**Figure 2A**). The second trend was a continuous decrease from C1 to C5 and included *IbMYB1* and *IbMYB4c*. The third trend was a continuous increase from C1 to C5 and included *IbMYB4b, Ib5GT1,* and *Ib5GT2*. The expression of the three MYB activators was very low in C4 and C5, which was consistent with the juvenile red fading phenomenon. Among the five MYB repressors, *IbMYB4a*, *IbMYB27,* and *IbMYBx* exhibited the first major trend, the expression of *IbbHLH2* was also significantly consistent with this trend, and the expression of *IbWDR1* was relatively stable with slight turbulence.

To further investigate the occurrence of any correlation in gene expression among the three pigment biosynthetic pathways, the relative expression trends of the structural genes for anthocyanins, chlorophylls, and carotenoids were obtained (**Supplementary Figure S4**) and the Pearson's correlation coefficients calculated. As it is shown in the **Supplementary Figure S5**, a significant correlation between the gene expression trends of the three pigment biosynthetic pathways did not emerge.

### Functional Verifications of the MBW Complexes and the MYB Repressors by Promoter Activation Tests

To further confirm the activities of the screened MYBs, promoter activation tests were conducted by dual luciferase assays. Tested TFs included eight MYBs, one bHLH (IbbHLH2), and one WDR (IbWDR1). The eight MYBs included three R2R3 activators (IbMYB1, IbMYB2, and IbMYB3), three AtMYB4-like R2R3 repressors (IbMYB4a, IbMYB4b, and IbMYB4c), one FaMYB1-like R2R3 repressor (IbMYB27), and one R3 repressor (IbMYBx). Constructed promoters included *pCHSD-555*, *pCHSE-1069*, *pDFRB-308*, *pbHLH2-892*, *pMYB27-768,* and *pMYB4c-805*. Among them, *pCHSD-555* and *pCHSE-1069* represented the upstream anthocyanin genes, *pDFRB-308* represented downstream genes, *pbHLH2-892* represented the bHLH of the MBW complex itself, and *pMYB27-768* and *pMYB4c-805* represented the promoters for FaMYB1-like and AtMYB4-like repressors.

**Figure 7:**
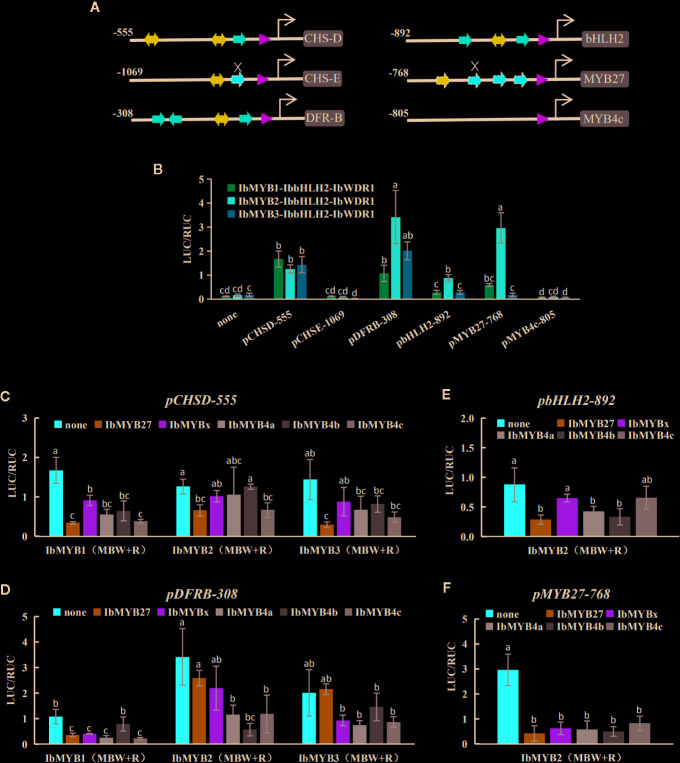
Responsiveness of the promoters to the MBW complexes and the MYB repressors tested by dual luciferase assays. **(A)** Proximal promoter architectures of the six cloned promoters. The numbers indicate the lengths of the cloned promoters. MREs and BREs are indicated with red and blue arrows, respectively. The TATA boxes are marked with green triangles. The *cis* motifs of *pCHSD-555*, *pDFRB-308*, and *pbHLH2-892* were indicated with solid arrows, as they were almost identical to their *I. purpurea* homologies reported by Zhu et al. (2015). The promoter of *pMYB27-768* was cloned in this study, and the dashed lines show the putative *cis* elements. Mutated MREs are indicated by the crosses. No MRE nor BRE were found in *pMYB4c-805*. **(B)** The activation of the MBW complexes on the six promoters. The X-axis shows the promoters and the Y-axis shows the LUC/RUC levels, with the error bars based on three biological replicates. Significant differences among means (p < 0.05) are indicated by letters (a, b, c, and d) above the bars by one-way ANOVA. **(C–F)** The responses of *pCHSD-555*, *pDFRB-308*, *pbHLH2-892*, and *pMYB27-768* after the addition of the five MYB repressors. The X-axes show the TF combinations and the Y-axes show the LUC/RUC levels, with the error bars based on three biological replicates. Significant differences among means (p < 0.05) are indicated by letters (a, b, and c) above the bars by one-way ANOVA.

The promoter sequences were first analyzed for their potential *cis* elements of the MYB-recognizing elements (MREs) and bHLH recognizing elements (BREs) for the anthocyanin MBW complexes (**Figure 7A**; **Supplementary Figure S6**) according to Zhu et al. (2015). Except for *pMYB4c-805*, putative MREs and BREs were found for the other five promoters. The sequences of *pCHSD-555*, *pDFRB-308,* and *pbHLH2-892* were highly homologous with those of *I. purpurea* (**Supplementary Figures S4A–C**)*, as* reported by Zhu et al. (2015). The promoter *pCHSD-555* and *pMYB27-768* seemed to have mutated MREs (**Figure 7A**, indicated by the crosses; **Supplementary Figure S6**).

The activation of the MBW complexes for each promoter was tested (**Figure 7B**). *pCHSE-1069* and *pMYB4c-805* were not activated at all, while the other four promoters could be activated by the MBW complexes at varied degrees. *pCHSD-555* responded similarly to the three MYB activators, while *pDFRB-308*, *pbHLH2-892,* and *pMYB27-768* responded the strongest to the IbMYB2-contained MBW complex.

The promoter activation results obtained after the addition of MYB repressors were shown in **Figures 7C–F**. Generally, the addition of any of the five inhibitors reduced the responsiveness of promoters, but the extent of this reduction varied. The overall reduction of *pCHSD-555* was not large, with the addition of IbMYB27 being the most effective. For the other three responsive promoters, IbMYB4b effectively inhibited the IbMYB2-contained MBW complex, but not so much for IbMYB1- or IbMYB3-contained MBW complexes. *pbHLH2-892* was activated at low levels by the MBW complexes, and the inhibitory effects were not that significant. *pMYB27-768* was strongly activated by the IbMYB2-contained MBW complex, and the addition of any of the five inhibitors could reduce the response to background levels.

### Regulatory Model of the MBW Complexes by Combining the Expression Profile and MYB Functions.

**Figure 8:**
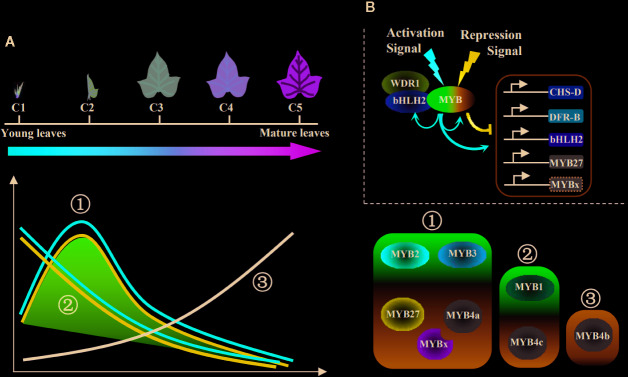
Regulatory model of the MBW complexes for juvenile red fading in sweetpotato leaves. **(A)** Three main trends summarized for the profiles of anthocyanin metabolites and underlying genes. The purple shadow represents the anthocyanin accumulation and expression of most structural genes, which followed the first major trend. The lines represent expressions of the eight MYBs, with all three trends included. Red lines represent MYB activators, blue lines represent accompanying MYB repressors, and the black line represents the antagonistic MYB repressor. Detailed trend classifications were shown on the right side for the MYB activators (purple shaded) and repressors (blue shaded). **(B)** Regulatory model of the MBW complexes involving multiple MYB activators and repressors. The signs above the MBW complex show possible hierarchical signals. The arrows below the MBW complex show the regulation on the anthocyanin pathway genes and possible feedback modulation within the MBW complexes. The red and blue colors represent activation and repression, respectively.

After combining data of gene expression profiles and promoter activation assays, the possible regulatory mechanisms were summarized in **Figure 8**. As the leaves developed, anthocyanin contents reached a peak at C2 and then decreased in the later stages. This trend (defined as the first major trend) was observed in the expression of most structural and regulatory genes (**Figure 6C**); however, two minor trends of continuous decreasing (the second trend) or increasing (the third trend) also occurred. The three trends were all reflected by the eight cloned MYBs (**Figure 6C**; **Figure 8A**). The first major trend included two activators (IbMYB2 and IbMYB3) accompanied by three repressors (IbMYB4a, IbMYB27, and IbMYBx). The second trend included one activator (IbMYB1) accompanied by one repressor (IbMYB4c). The third trend contained only one repressor (IbMYB4b), which significantly inhibited three tested promoters (*pDFRB-308*, *pbHLH2-892,* and *pMYB27-768*). Among the five repressors, four were accompanying repressors expressed along with MYB activators, indicating their roles in limiting the production of anthocyanins once activated by endogenous or exogenous signals. The only antagonistic MYB repressor (IbMYB4b, identified in the third trend) may be crucial for the decrease in anthocyanin content observed during the later stages of leaf development.

The above data implies that multiple MYB activators and repressors work collaboratively to regulate the juvenile red fading in leaves of sweetpotato, involving both hierarchical and feedback regulation of the MBW complexes (**Figure 8B**). The feedback regulation may fine-tune the regulation of anthocyanin accumulation *via* the MYB activators and repressors (possibly through MYBs in the first major trend). Hierarchical signals may function in the initiation (possibly through MYBs in the second trend) and termination (possibly through the MYB in the third trend) of the anthocyanin biosynthetic pathway.

## Discussion

The juvenile red fading phenomenon is commonly observed in woody plants, especially in tropical areas, but occurs less often in herbaceous plants (e.g., the classic model plants Arabidopsis, maize, rice, and petunia). This study has provided a comprehensive analysis of the metabolites and molecular mechanisms of the anthocyanin pathway responsible for juvenile red fading in the herbaceous vine plant sweetpotato. A network of MBW complexes including multiple MYB activators and repressors was proposed to participate in the fine-tuning of the juvenile red fading process.

### Consistent Trends for Anthocyanin Accumulation and Expressions of Anthocyanin Genes

The accumulation of anthocyanins displayed a peak at stage C2; then it decreased at the later stages, as it can be evinced from spectrophotometric measurements (**Figure 1B**), microscopic leaf sections (**Figure 1C**), and quantification by UPLC-MS/MS (**Figure 2A**). Incidentally, this trend is not easy to be seen by bare eyes as the red leaf epidermis masks the coloration of the mesophyll beneath as well as the presence of chlorophylls in this tissue (**Figure 1A**). The difference in anthocyanin content between adjacent stages was, in fact, significant in the C2-vs-C3 comparison according to both spectrophotometric and UPLC-MS/MS analyses, and C3-vs-C4 comparison but by spectrophotometric analysis only. Thus, from stages C3 to C5, the changes of anthocyanins appeared to be not that drastic (**Figure 1B**; **Figure 2**).

Although the trend in gene expression profile monitored either by RNA-seq (**Supplementary Figure S5**) or qRT-PCR (**Figure 6C**) was quite consistent with that of anthocyanin accumulation (**Figure 2**), some differences concerning gene expression data emerged between the two methods. While RNA-seq data showed that the flavonoid/anthocyanin genes were mainly enriched in the DEG groups of C3-vs-C4 and C4-vs-C5 (**Table 2**), qRT-PCR evidenced that the expression levels of some activators (*IbMYB1*, *IbMYB3*, and *IbbHLH2*) and structural genes of anthocyanins (*IbF3H*, *IbF3'H*, *IbANS*, and *IbGGT1*) significantly decreased between stages C2 and C3, when also the levels of anthocyanins dropped. Additionally, by DEG analysis for adjacent leaf stages, thousands of genes were enriched in each of our comparisons, but several key genes such as *IbMYB2*, *IbMYB4b*, and *IbMYB4c* were not identified in any of the comparison groups (**Table 2**). Comparison for non-adjacent stages, such as C2-vs-C5, may include these genes into the DEG list. Conversely, from qRT-PCR data it emerged that genes such as *IbMYB4b* and *IbMYB4c* were differentially expressed between some adjacent leaf stages. These discrepancies are consistent with the observation that while RNA-seq can be used to analyze the differences in gene expression on a large scale, reflecting the overall trend of gene expression in the samples, qRT-PCR remains the method of choice for gene expression analyses as well for the validation of data obtained by high-throughput profiling methods (Robert and Watson, 2015; Everaert et al., 2017).

### The Anthocyanin Genes Expressed in Sweetpotato Leaves were Comparable with those in Morning Glories

The genomic sequences of *I. batata*s, a hexaploid species with 90 chromosomes, were released based on the haplotype of 15 chromosomes for the cultivar “Taizhong 6” (Yang et al., 2017). Hexaploidy and the great genetic diversity among sweetpotato cultivars may have contributed to the low mapping ratio of “Chuanshan Zi” to “Taizhong 6”, which was approximately 72%. The alignment showed much incompleteness and inaccuracy, with many degenerate bases and rare codons (e.g., “UGA” for selenocysteine) in the CDS database. No MYBs from the genome of “Taizhong 6” clustered with the Arabidopsis SG6 MYBs (data not shown). The screened anthocyanin- unigenes were blasted against the reference genome. With the criteria of E-value < 1×10^-5^, most of the unigenes can be assigned to multiple locations, possibly as a result of the hexaploid state of the genome. Three of the confirmed genes, including *IbMYB3*, *IbWDR1*, and *IbWDR2*, cannot be blasted to any locations (**Supplementary Table S2**). The alignment to the “Taizhong 6” reference genome was used only as auxiliary data for subsequent cloning of the promoters. However, the screened structural and regulatory genes for the anthocyanin pathway were highly comparable with those reported in its diploid relatives *I. purpurea* and *I. nil*. We considered that the anthocyanin genes may have undergone rigorous dosage selection to maintain their copy numbers. The unique red juvenile leaves in multiple sweetpotato cultivars were absent in the diploid morning glories, implying that this phenotype acquisition event might be related to the genome triplication.

The promoters of *CHS* appear to be very conserved among species, including both monocots and dicots (**Supplementary Figure S7**). They typically possess BRE (CACGTG and variants) and MRE [A(G/C/A)CTACC] located in close proximity just upstream of the TATA box. However, *IbCHS-E* expressed differently (**Figure 6C**) and *pCHSE-1069* was unresponsive in the promoter activation assays (**Figure 7B**). This result was consistent with the research of Morita et al. (2006), who reported that all anthocyanin structural genes except *InCHS-E* were down-regulated in the *wdr1* mutant of *I. nil*. Sequence examination of *pCHSE-1069* showed that its MRE was mutated at the center (AGCTgCC), which may explain its unresponsiveness to MBW complexes. But the variant AGCTgCC motif may respond to other flavonoid-MYBs. In addition, the 5'UTR of *pCHSE-1069* is much longer than that of other anthocyanin genes, which may also be the reason for its failure to respond to the MBW complexes.

Among the regulatory genes, *IbbHLH2* and *IbWDR1* were comparable with those expressed in flowers of *I. nil* (*InbHLH2* and *InWDR1)*. Three MYB activators, corresponding to *InMYB1*, *InMYB2*, and *InMYB3* (Morita et al., 2006), were screened and cloned in our study. The three MYBs of *I. nil* showed tissue-specific expression: *InMYB1* was expressed in flowers, *InMYB2* was expressed in the vegetative tissue, and the expression of *InMYB3* was not undetected (Morita et al., 2006). Our results indicate that all the three MYBs play active roles in anthocyanin accumulation in sweetpotato leaves. Our cloned unigene0031238/*IbMYB1* has the identical CDS sequence as the *IbMYB1* reported for purple-fleshed sweetpotato roots in Mano et al. (2007), although no associated MYBs were found in red leaves by their RT-PCR assays. The gene expression profiles in our study provide some clues to the observed paradox: although leaves at stage C3 were young and red, the expression of the three MYB activators had already dropped to extremely low levels, especially for *IbMYB1* and *IbMYB3*, which may hinder them from being detected by RT-PCR.

### Multiple MYB Activators and Repressors Collaboratively Regulate the Process of Juvenile Red Fading in Sweetpotato Leaves

MYBs were proposed to be the key factors of the MBW complexes in many species, determining the quantities and spatio-temporal accumulation of anthocyanins. Our study reports that three MYB activators and five MYB repressors are involved in the process of juvenile red fading in sweetpotato leaves. Two regulation modes for MYB repressors were involved: (1) R2R3- or R3-type MYB repressors simultaneously expressed with MYB activators and (2) a single antagonistic repressor (IbMYB4b) which may be the key for the decline of anthocyanin content at later stages of leaf development.

Notably, all three MYB activators were accompanied by simultaneously expressed MYB repressors. Similar cases were reported in petunia, whereby the expression of the repressors *PhMYB27* and *PhMYBx* were induced by the MBW complexes in both leaves and flowers (Albert et al., 2014). The feedback regulation that we described has also been found in a study of forage legumes (*Trifolium repens*), which reported that the anthocyanin- and PA-related MYB activators induced the MYB repressors Tr-MYB133 and Tr-MYB134, thereby inhibiting the expression of *TrDFR* and *TrANR* (Albert, 2015). Similarly, in a study of peach fruit (*Prunus persica*), the repressor *PpMYB18* was highly expressed during anthocyanin and procyanidin accumulation and seemed to be induced by anthocyanin- and PA-related MYB activators (Zhou et al., 2019). These accompanying MYB repressors from various species were assumed to prevent excessive accumulation of anthocyanins in plant tissues. In our study, the only antagonistic repressor, IbMYB4b, was possibly the key transcription factor underlying the red fading phenomenon observed at later stages of sweetpotato leaf development.

Albert et al. (2014) proposed mechanisms of hierarchical and feedback regulation involving activators and repressors that may function widely in dicots. This proposed regulatory model was supported by our findings: hierarchical regulation may be used in signal perceptions and feedback regulation may be used for the fine-tuning of anthocyanins. It is reasonable to speculate that IbMYB1 (within the second trend, accompanied by IbMYB4c) might be the critical initiation MYB of the anthocyanin pathway, while IbMYB2 and IbMYB3 (within the first trend, accompanied by IbMYB27, IbMYB4a, and IbMYBx) were possibly the maintaining MYB activators. IbMYB4b (within the third trend) was likely crucial for the termination of anthocyanin pathway when the leaves were nearing maturity. From the promoter activation assays, we determined that all MBW-responsive promoters corresponded to genes within the first major trend and may function in the way of a feedback system. Promoters for IbMYB4c and IbMYB4b were more likely stimulated by hierarchical developmental signals rather than the MBW complex itself.

This study explored the elaborate regulatory network of anthocyanins responsible for the juvenile red fading phenomenon observed in the herbaceous vine plant sweetpotato. It was determined that the collaborative regulation of multiple MYBs allowed sweetpotato leaves to accumulate anthocyanins in a timely manner according to their stage of development. The red fading process protects the young leaves while ensuring that later photosynthetic capacity is not hindered. This study furthered our understanding of the spatio-temporal accumulation of anthocyanins and the revealed molecular mechanism underlying the sweetpotato juvenile red fading process may also provide clues for similar studies in other plant species.

## Data Availability Statement

The datasets generated for this study can be found in the NCBIShort Read Archive No. PRJNA612413, and under GenBank accession numbers of MT231489, MT231490, MT231491, MT231492, MT231493, MT231494, MT231495, MT231496, MT231497, MT231498, MT231499, MT231500, MT231501, MT231502, MT231503, MT231504, MT557573, MT557574, MT557575, MT557576, MT557577, MT557578, MT557579, MT557580, MT557581, MT557582, MT557583, and MT557584.

## Author Contributions

ZZ, KB, HW, GZ, YL, and JW contributed to project planning and data analysis. GZ provided the sweetpotato germplasm. JD and DW performed the transcriptomic analysis and managed the experiments. DW and JS performed the dual luciferase assays. ZZ wrote the manuscript. KB, HW, and JD contributed to the proofreading of this manuscript. All authors read and approved the final manuscript.

## Funding

This work was financially supported by the National Natural Science Foundation of China (31660074 and 31660055) and the Natural Science Foundation of Hainan Province (317012).

## Conflict of Interest

The authors declare that the research was conducted in the absence of any commercial or financial relationships that could be construed as a potential conflict of interest. 

## Abbreviations

BRE, bHLH recognizing element; DEGs, differentially expressed genes; MBW complex, MYB-bHLH-WDR complex; MRE, MYB-recognizing element; TFs, transcription factors.

## References

[B1] Aharoni A. De Vos C. H. R. Wein M. Sun Z. K. Greco R. Kroon A. (2001). The strawberry *FaMYB1* transcription factor suppresses anthocyanin and flavonol accumulation in transgenic tobacco. Plant J. 28 (3), 319–332. 10.1046/j.1365-313X.2001.01154.x 11722774

[B2] Albert N. W. Lewis D. H. Zhang H. Schwinn K. E. Jameson P. E. Davies K. M. (2011). Members of an R2R3-MYB transcription factor family in *Petunia* are developmentally and environmentally regulated to control complex floral and vegetative pigmentation patterning. Plant J. 65 (5), 771–784. 10.1111/j.1365-313X.2010.04465.x 21235651

[B3] Albert N. W. Davies K. M. Lewis D. H. Zhang H. Montefiori M. Brendolise C. (2014). A conserved network of transcriptional activators and repressors regulates anthocyanin pigmentation in eudicots. Plant Cell 26 (3), 962–980. 10.1105/tpc.113.122069 24642943 PMC4001404

[B4] Albert N. W. (2015). Subspecialization of R2R3-MYB repressors for anthocyanin and proanthocyanidin regulation in forage Legumes. Front. Plant Sci. 6, 1165. 10.3389/fpls.2015.01165 26779194 PMC4689181

[B5] Borevitz J. O. Xia Y. J. Blount J. Dixon R. A. Lamb C. (2000). Activation tagging identifies a conserved MYB regulator of phenylpropanoid biosynthesis. Plant Cell 12 (12), 2383–2393. 10.1105/tpc.12.12.2383 11148285 PMC102225

[B6] Cao X. Qiu Z. Wang X. Van Giang T. Liu X. Wang J. (2017). A putative *R3 MYB* repressor is the candidate gene underlying *atroviolacium*, a locus for anthocyanin pigmentation in tomato fruit. J. Exp. Bot. 68 (21-22), 5745–5758. 10.1093/jxb/erx382 29186488 PMC5854135

[B7] Carey C. C. Strahle J. T. Selinger D. A. Chandler V. L. (2004). Mutations in the *pale aleurone color1* regulatory gene of the *Zea mays* anthocyanin pathway have distinct phenotypes relative to the functionally similar *TRANSPARENT TESTA GLABRA1* gene in *Arabidopsis thaliana* . Plant Cell 16 (2), 450–464. 10.1105/tpc.018796 14742877 PMC341916

[B8] Cavallini E. Matus J. T. Finezzo L. Zenoni S. Loyola R. Guzzo F. (2015). The phenylpropanoid pathway is controlled at different branches by a set of R2R3-MYB C2 repressors in grapevine. Plant Physiol. 167 (4), 1448–1470. 10.1104/pp.114.256172 25659381 PMC4378173

[B9] Chen Y. Z. Huang S. Q. (2013). Red young leaves have less mechanical defence than green young leaves. Oikos 122, 1035–1041. 10.1111/j.1600-0706.2012.20852.x

[B10] Chiu L.-W. Li L. (2012). Characterization of the regulatory network of BoMYB2 in controlling anthocyanin biosynthesis in purple cauliflower. Planta 236 (4), 1153–1164. 10.1007/s00425-012-1665-3 22644767

[B11] Davies K. M. Albert N. W. Schwinn K. E. (2012). From landing lights to mimicry: the molecular regulation of flower colouration and mechanisms for pigmentation patterning. Funct. Plant Biol. 39 (8), 619–638. 10.1071/fp12195 32480814

[B12] Des Marais D. L. Rausher M. D. (2008). Escape from adaptive conflict after duplication in an anthocyanin pathway gene. Nature 454 (7205), 762–765. 10.1038/nature07092 18594508

[B13] Dominy N. J. Lucas P. W. Ramsden L. W. Riba-Hernandez P. Stoner K. E. IM T. (2002). Why are young leaves red? Oikos 98, 163–176. 10.1034/j.1600-0706.2002.980117.x

[B14] Dong W. Niu L. Gu J. Gao F. (2014). Isolation of a WD40-repeat gene regulating anthocyanin biosynthesis in storage roots of purple-fleshed sweet potato. Acta Physiol. Plant 36 (5), 1123–1132. 10.1007/s11738-014-1487-y

[B15] Drumm-Herrel H. Mohr H. (1982). Effect of blue/UV light on anthocyanin synthesis in tomato seedlings in the absence of bulk carotenoids. Photochem. Photobiol. 36, 229–233. 10.1111/j.1751-1097.1982.tb04368.x

[B16] Dubos C. Stracke R. Grotewold E. Weisshaar B. Martin C. Lepiniec L. (2010). MYB transcription factors in *Arabidopsis* . Trends Plant Sci. 15 (10), 573–581. 10.1016/j.tplants.2010.06.005 20674465

[B17] Durbin M. L. Denton A. L. Clegg M. T. (2001). Dynamics of mobile element activity in chalcone synthase loci in the common morning glory (*Ipomoea purpurea*). Proc. Natl. Acad. Sci. U. S. A 98 (9), 5084–5089. 10.1073/pnas.091095498 11309503 PMC33167

[B18] Espley R. V. Brendolise C. Chagne D. Kutty-Amma S. Green S. Volz R. (2009). Multiple repeats of a promoter segment causes transcription factor autoregulation in red apples. Plant Cell 21 (1), 168–183. 10.1105/tpc.108.059329 19151225 PMC2648084

[B19] Everaert C. Luypaert M. Maag J. L. V. Cheng Q. X. Dinger M. E. Hellemans J. (2017). Benchmarking of RNA-sequencing analysis workflows using whole-transcriptome RT-qPCR expression data. Sci. Rep. 7 (1), 1559. 10.1038/s41598-017-01617-3 28484260 PMC5431503

[B20] Gong W. C. Liu Y. H. Wang C. M. Chen Y. Q. Martin K. Meng L. Z. (2020). Why are there so many plant species that transiently flush young leaves red in the tropics? Front. Plant Sci. 11, 83. 10.3389/fpls.2020.00083 PMC7041174 32133020

[B21] Gould K. S. (2004). Nature's Swiss Army Knife: The Diverse Protective Roles of Anthocyanins in Leaves. J. Biomed. Biotechnol. 2004 (5), 314–320. 10.1155/S1110724304406147 15577195 PMC1082902

[B22] Hichri I. Barrieu F. Bogs J. Kappel C. Delrot S. Lauvergeat V. (2011). Recent advances in the transcriptional regulation of the flavonoid biosynthetic pathway. J. Exp. Bot. 62 (8), 2465–2483. 10.1093/jxb/erq442 21278228

[B23] Hoshino A. Jayakumar V. Nitasaka E. Toyoda A. Noguchi H. Itoh T. (2016). Genome sequence and analysis of the Japanese morning glory *Ipomoea nil* . Nat. Commun. 7, 13295. 10.1038/ncomms13295 27824041 PMC5105172

[B24] Hughes N. M. Morley C. B. Smith W. K. (2007). Coordination of anthocyanin decline and photosynthetic maturation in juvenile leaves of three deciduous tree species. New Phytol. 175 (4), 675–685. 10.1111/j.1469-8137.2007.02133.x 17688583

[B25] Hughes N. M. Vogelmann T. C. Smith W. K. (2008). Optical effects of abaxial anthocyanin on absorption of red wavelengths by understorey species: revisiting the back-scatter hypothesis. J. Exp. Bot. 59 (12), 3435–3442. 10.1093/jxb/ern193 18653695 PMC2529231

[B26] Hughes N. M. Carpenter K. L. Keidel T. S. Miller C. N. Waters M. N. Smith W. K. (2014). Photosynthetic costs and benefits of abaxial versus adaxial anthocyanins in *Colocasia esculenta* ‘Mojito'. Planta 240 (5), 971–981. 10.1007/s00425-014-2090-6 24903360

[B27] Karageorgou P. Manetas Y. (2006). The importance of being red when young: anthocyanins and the protection of young leaves of *Quercus coccifera* from insect herbivory and excess light. Tree Physiol. 26 (5), 613–621. 10.1093/treephys/26.5.613 16452075

[B28] Kumar S. Stecher G. Li M. Knyaz C. Tamura K. (2018). MEGA X: Molecular Evolutionary Genetics Analysis across Computing Platforms. Mol. Biol. Evol. 35 (6), 1547–1549. 10.1093/molbev/msy096 29722887 PMC5967553

[B29] Luo J. Duan J. Huo D. Shi Q. Niu L. Zhang Y. (2017). Transcriptomic analysis reveals transcription factors related to leaf anthocyanin biosynthesis in *Paeonia qiui* . Molecules 22 (12), 2186. 10.3390/molecules22122186 PMC6149671 29292771

[B30] Ma D. Constabel C. P. (2019). MYB repressors as regulators of phenylpropanoid metabolism in plants. Trends Plant Sci. 24 (3), 275–289. 10.1016/j.tplants.2018.12.003 30704824

[B31] Ma D. Reichelt M. Yoshida K. Gershenzon J. Constabel C. P. (2018). Two R2R3-MYB proteins are broad repressors of flavonoid and phenylpropanoid metabolism in poplar. Plant J. 96 (5), 949–965. 10.1111/tpj.14081 30176084

[B32] Mano H. Ogasawara F. Sato K. Higo H. Minobe Y. (2007). Isolation of a regulatory gene of anthocyanin biosynthesis in tuberous roots of purple-fleshed sweet potato. Plant Physiol. 143 (3), 1252–1268. 10.1104/pp.106.094425 17208956 PMC1820918

[B33] Matus J. T. Poupin M. J. Canon P. Bordeu E. Alcalde J. A. Arce-Johnson P. (2010). Isolation of WDR and bHLH genes related to flavonoid synthesis in grapevine (*Vitis vinifera* L.). Plant Mol. Biol. 72 (6), 607–620. 10.1007/s11103-010-9597-4 20112051

[B34] Morita Y. Saitoh M. Hoshino A. Nitasaka E. Iida S. (2006). Isolation of cDNAs for R2R3-MYB, bHLH and WDR transcriptional regulators and identification of *c* and *ca* mutations conferring white flowers in the Japanese morning glory. Plant Cell Physiol. 47 (4), 457–470. 10.1093/pcp/pcj012 16446312

[B35] Ording E. Kvavik W. Bostad A. Gabrielsen O. S. (1994). Two functionally distinct half sites in the DNA-recognition sequence of the Myb oncoprotein. Eur. J. Biochem. 222 (1), 113–120. 10.1111/j.1432-1033.1994.tb18848.x 8200335

[B36] Park K. I. Ishikawa N. Morita Y. Choi J. D. Hoshino A. Iida S. (2007). A bHLH regulatory gene in the common morning glory, *Ipomoea purpurea*, controls anthocyanin biosynthesis in flowers, proanthocyanidin and phytomelanin pigmentation in seeds, and seed trichome formation. Plant J. 49 (4), 641–654. 10.1111/j.1365-313X.2006.02988.x 17270013

[B37] Pfaffl M. W. (2001). A new mathematical model for relative quantification in real-time RT-PCR. Nucleic Acids Res. 29, 2002–2007. 10.1093/nar/29.9.e45 PMC55695 11328886

[B38] Pires N. Dolan L. (2010). Origin and diversification of basic-helix-loop-helix proteins in plants. Mol. Biol. Evol. 27 (4), 862–874. 10.1093/molbev/msp288 19942615 PMC2839125

[B39] Ramsay N. A. Glover B. J. (2005). MYB-bHLH-WD40 protein complex and the evolution of cellular diversity. Trends Plant Sci. 10 (2), 63–70. 10.1016/j.tplants.2004.12.011 15708343

[B40] Robert C. Watson M. (2015). Errors in RNA-Seq quantification affect genes of relevance to human disease. Genome Biol. 16, 177. 10.1186/s13059-015-0734-x 26335491 PMC4558956

[B41] Salvatierra A. Pimentel P. Alejandra Moya-Leon M. Herrera R. (2013). Increased accumulation of anthocyanins in *Fragaria chiloensis* fruits by transient suppression of *FcMYB1* gene. Phytochemistry 90, 25–36. 10.1016/j.phytochem.2013.02.016 23522932

[B42] Schwinn K. E. Ngo H. Kenel F. Brummell D. A. Albert N. W. McCallum J. A. (2016). The onion (*Allium cepa* L.) R2R3-MYB gene MYB1 regulates anthocyanin biosynthesis. Front. Plant Sci. 7, 1865. 10.3389/fpls.2016.01865 28018399 PMC5146992

[B43] Stracke R. Jahns O. Keck M. Tohge T. Niehaus K. Fernie A. R. (2010). Analysis of PRODUCTION OF FLAVONOL GLYCOSIDES-dependent flavonol glycoside accumulation in *Arabidopsis thaliana* plants reveals MYB11-, MYB12-and MYB111-independent flavonol glycoside accumulation. New Phytol. 188 (4), 985–1000. 10.1111/j.1469-8137.2010.03421.x 20731781

[B44] Sun B. Zhu Z. Cao P. Chen H. Chen C. Zhou X. (2016). Purple foliage coloration in tea (*Camellia sinensis* L.) arises from activation of the R2R3-MYB transcription factor CsAN1. Sci. Rep. 6, 32534. 10.1038/srep32534 27581206 PMC5007479

[B45] Wang H. Guan S. Zhu Z. Wang Y. Lu Y. (2013). A valid strategy for precise identifications of transcription factor binding sites in combinatorial regulation using bioinformatic and experimental approaches. Plant Methods 9 (1), 34. 10.1186/1746-4811-9-34 23971995 PMC3847620

[B46] Wu J. F. Tsai H. L. Joanito I. Wu Y. C. Chang C. W. Li Y. H. (2016). LWD-TCP complex activates the morning gene CCA1 in Arabidopsis. Nat. Commun. 7, 13181. 10.1038/ncomms13181 27734958 PMC5065627

[B47] Wu S. Lau K. H. Cao Q. Hamilton J. P. Sun H. Zhou C. (2018). Genome sequences of two diploid wild relatives of cultivated sweetpotato reveal targets for genetic improvement. Nat. Commun. 9 (1), 4580. 10.1038/s41467-018-06983-8 30389915 PMC6214957

[B48] Xu W. Grain D. Bobet S. Le Gourrierec J. Thevenin J. Kelemen Z. (2014). Complexity and robustness of the flavonoid transcriptional regulatory network revealed by comprehensive analyses of MYB-bHLH-WDR complexes and their targets in Arabidopsis seed. New Phytol. 202 (1), 132–144. 10.1111/nph.12620 24299194

[B49] Yang J. Moeinzadeh M. H. Kuhl H. Helmuth J. Xiao P. Haas S. (2017). Haplotype-resolved sweet potato genome traces back its hexaploidization history. Nat. Plants 3 (9), 696–703. 10.1038/s41477-017-0002-z 28827752

[B50] Zhou H. Lin-Wang K. Wang F. Espley R. V. Ren F. Zhao J. (2019). Activator-type R2R3-MYB genes induce a repressor-type R2R3-MYB gene to balance anthocyanin and proanthocyanidin accumulation. New Phytol. 221 (4), 1919–1934. 10.1111/nph.15486 30222199

[B51] Zhu Z. X. Wang H. L. Wang Y. T. Guan S. Wang F. Tang J. Y. (2015). Characterization of the *cis* elements in the proximal promoter regions of the anthocyanin pathway genes reveals a common regulatory logic that governs pathway regulation. J. Exp. Bot. 66 (13), 3775–3789. 10.1093/jxb/erv173 25911741 PMC4473980

[B52] Zhu H. Zhang T. J. Zheng J. Huang X. D. Yu Z. C. Peng C. L. (2018). Anthocyanins function as a light attenuator to compensate for insufficient photoprotection mediated by nonphotochemical quenching in young leaves of Acmena acuminatissima in winter. Photosynthetica 56 (1), 445–454. 10.1007/s11099-017-0740-1

[B53] Zimmermann I. M. Heim M. A. Weisshaar B. Uhrig J. F. (2004). Comprehensive identification of *Arabidopsis thaliana* MYB transcription factors interacting with R/B-like BHLH proteins. Plant J. 40 (1), 22–34. 10.1111/j.1365-313X.2004.02183.x 15361138

[B54] Zufall R. A. Rausher M. D. (2004). Genetic changes associated with floral adaptation restrict future evolutionary potential. Nature 428 (6985), 847–850. 10.1038/nature02489 15103375

