# Supplementary material for: Multiple MYB Activators and Repressors Collaboratively Regulate the Juvenile Red Fading in Leaves of Sweetpotato
Source: Front Plant Sci. 2020 Jun 25;11:941. doi: 10.3389/fpls.2020.00941 (PMC7330089; doi:10.3389/fpls.2020.00941)
Supplement: Supplementary file 1 [file DataSheet_1.pdf]

# Fig. S1

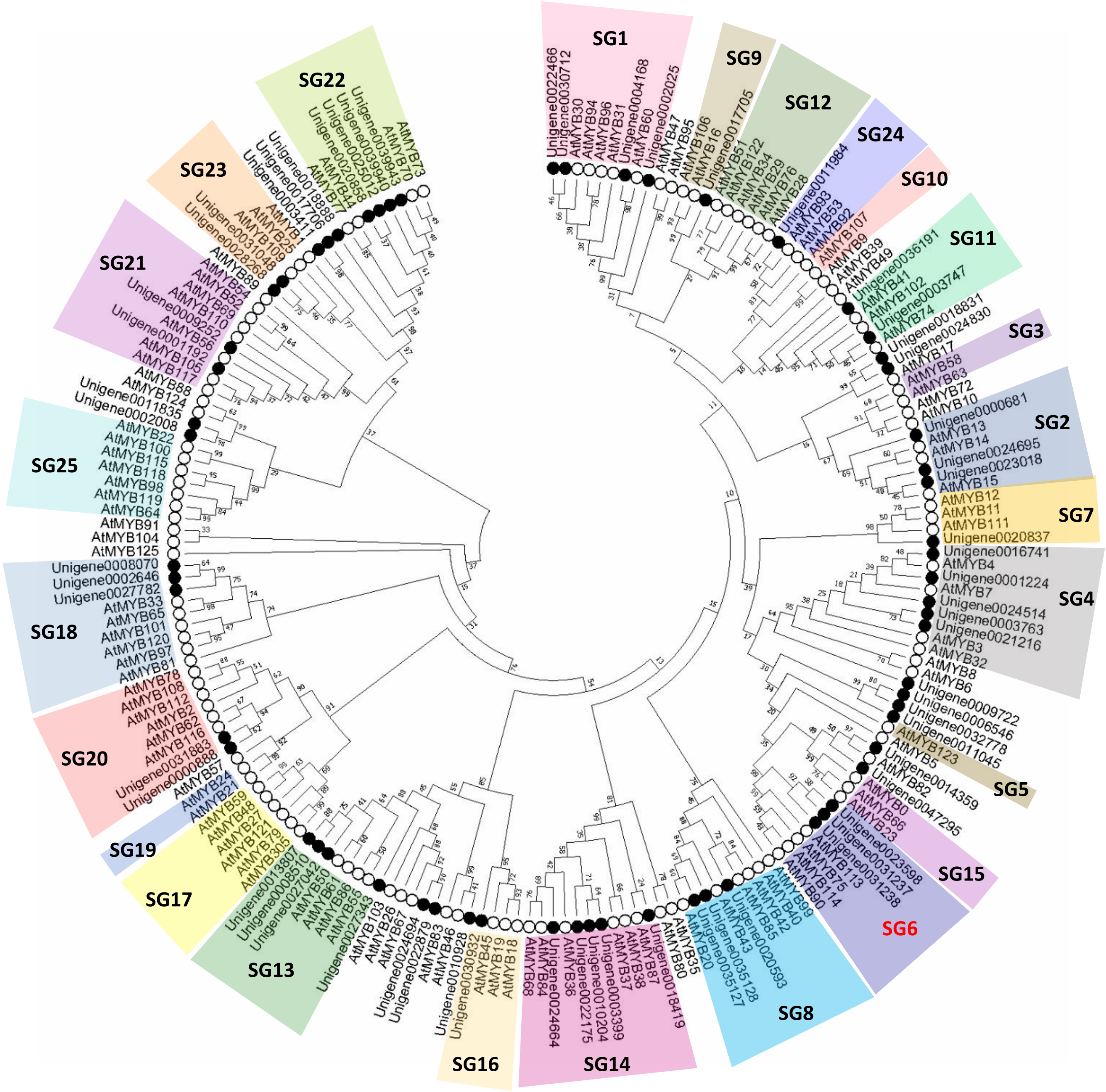


Fig. S1. Phylogenetic analysis of R2R3-MYBs from sweetpotato and Arabidopsis. Only the R2R3 domains were included in the analysis. The black dots show the sweetpotato unigenes and the white dots show the Arabidopsis R2R3-MYB members. The subgroup (SG) classification followed that of Dubos et al. (2010).

# Fig. S2

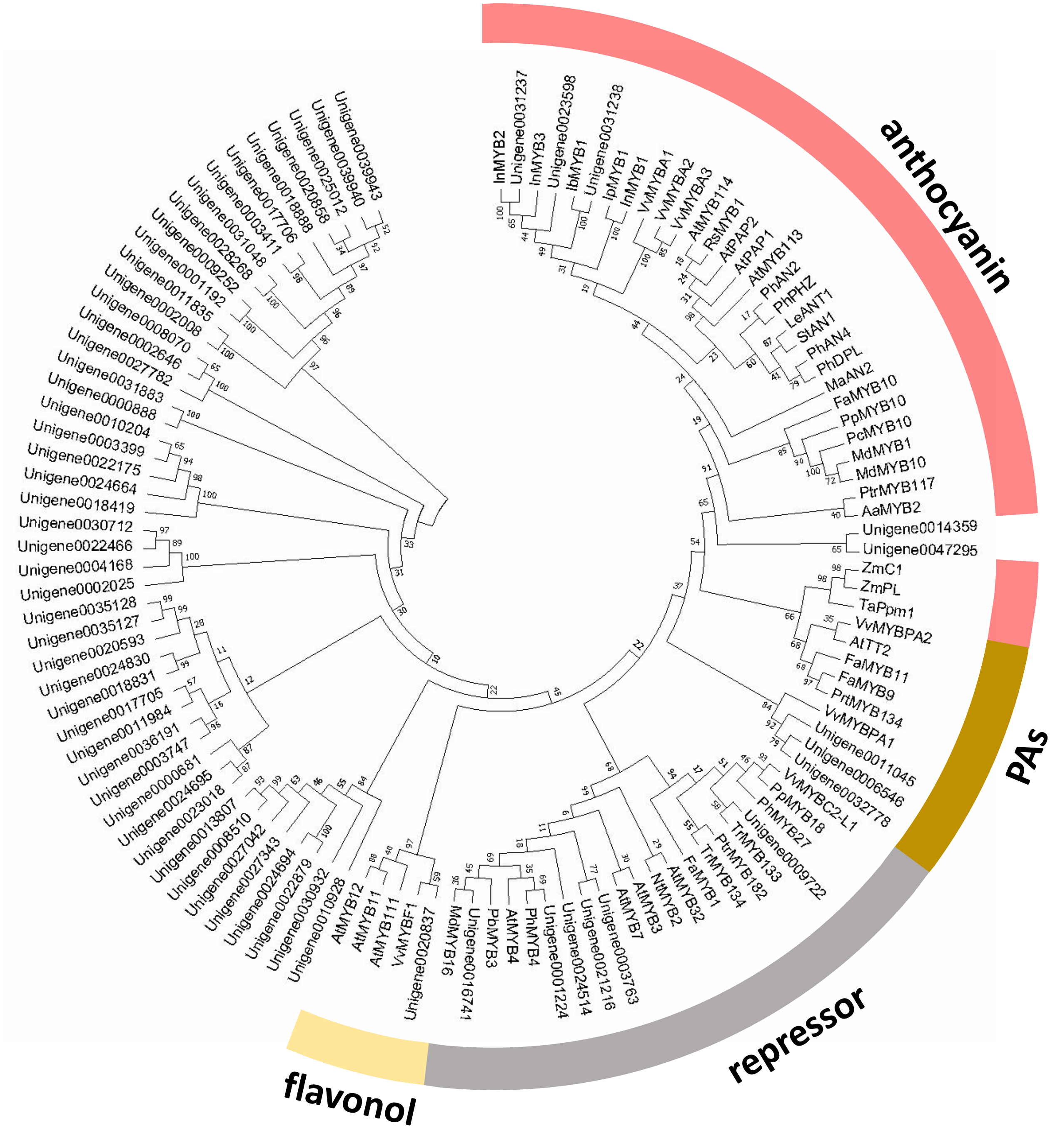


Fig. S2. Phylogenetic analysis of sweetpotato R2R3-MYBs with functional verified flavonoid-related MYBs in other species.

Only the R2R3 domains were included in the analysis. The red, brown, and yellow regions represent R2R3-MYB activators of anthocyanins, PAs, and flavonols, respectively, while the gray region represents inhibitory R2R3-MYBs.

# Fig. S3

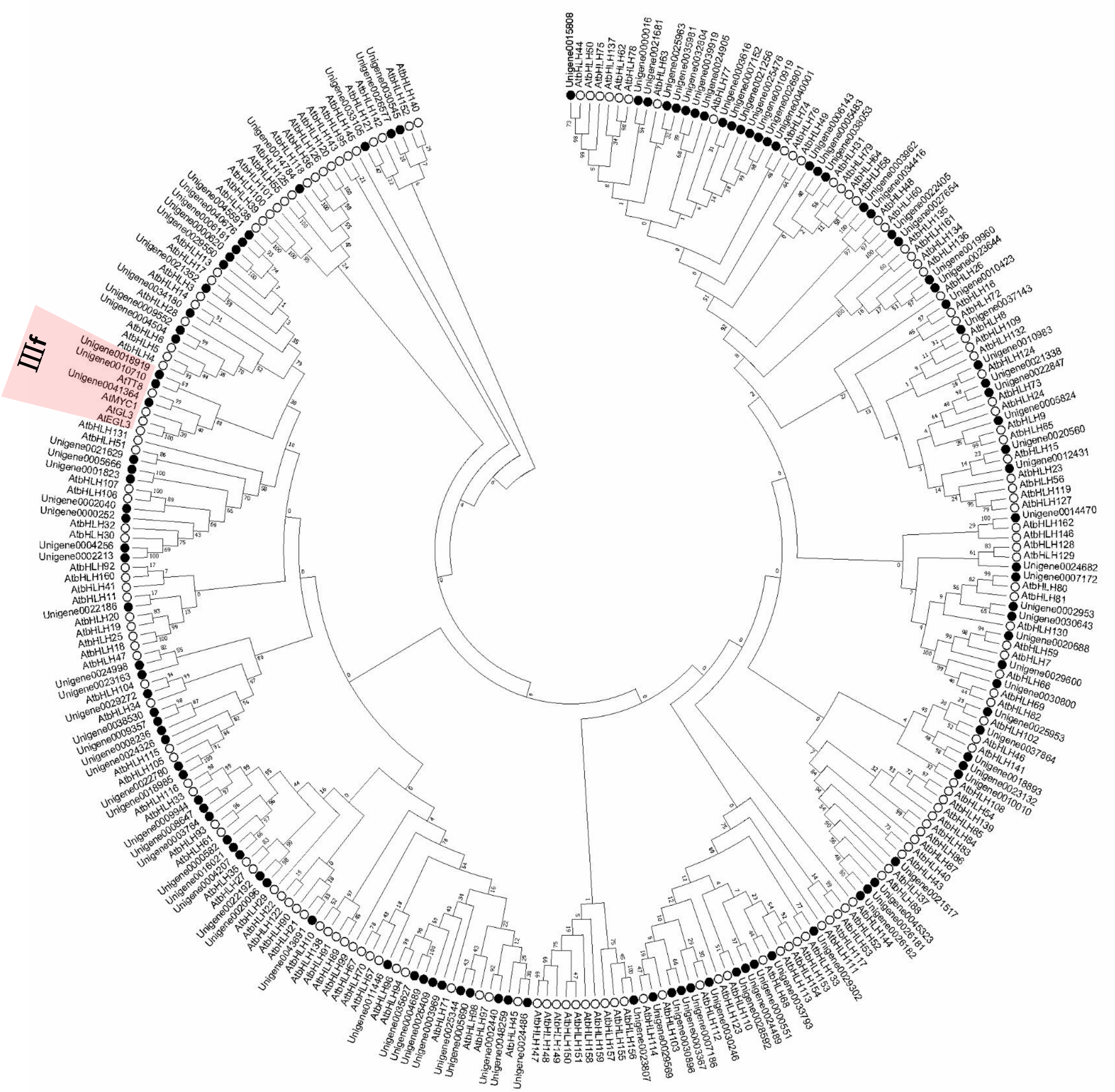


Fig. S3. Phylogenetic analysis of sweetpotato bHLHs with the Arabidopsis bHLH family. The whole protein sequences were included in the analysis. The red shade shows the SGIIIf subfamily involved in flavonoid regulation, the black dots show the sweetpotato unigenes, and the white dots show the Arabidopsis bHLH members.

# Fig. S4

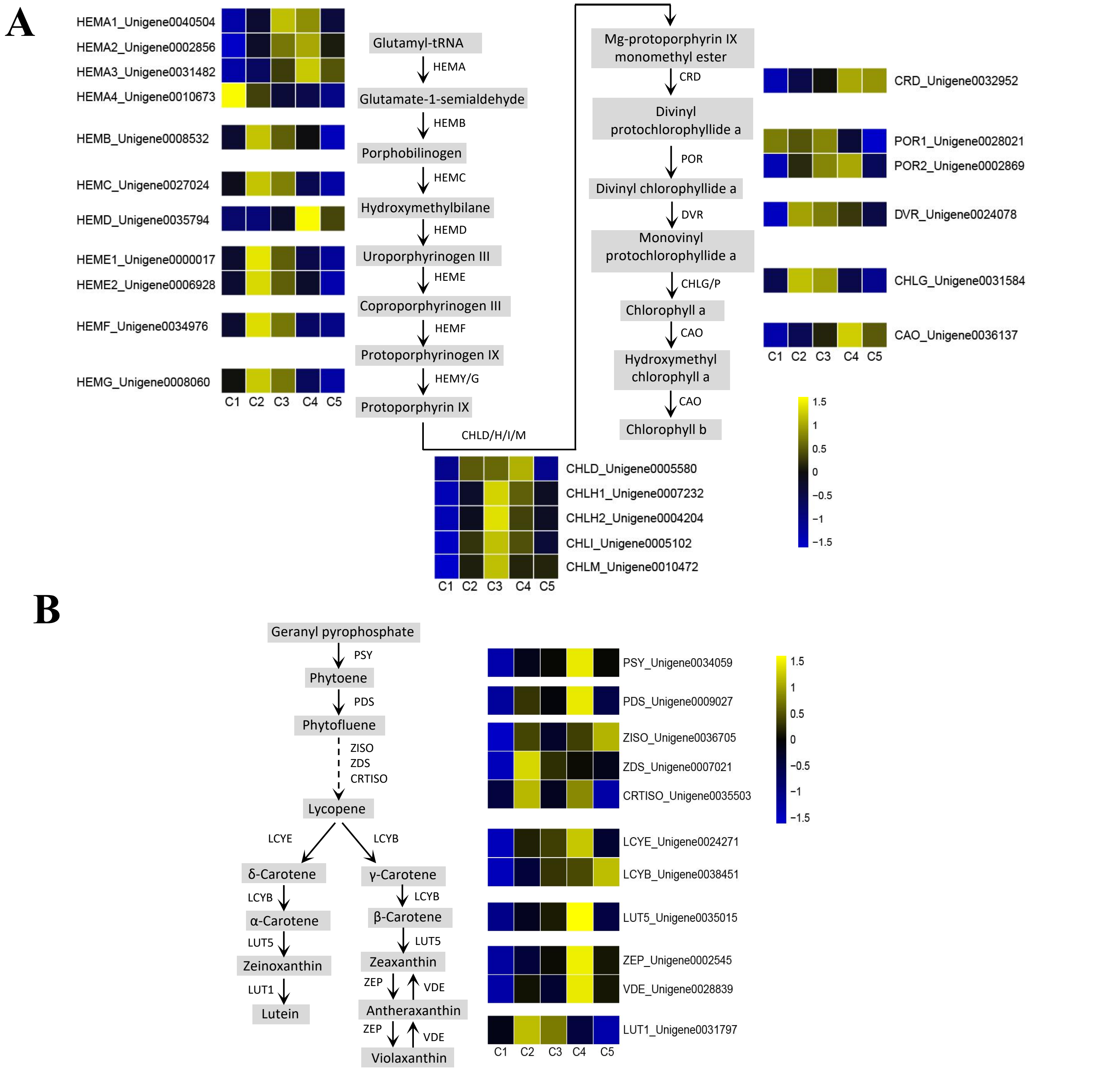


Fig. S4. The gene expressions for chlorophyll metabolism and carotenoid biosynthesis based on RPKMs. (A) Analysis for chlorophyll metabolism. (B) Analysis for carotenoid biosynthesis. HEMA, glutamyl-tRNA reductase; HEMB, porphobilinogen synthase; HEMC, hydroxymethylbilane synthase; HEMD, uroporphyrinogen-III synthase; HEME, uroporphyrinogen decarboxylase; HEMF, coproporphyrinogen III oxidase; HEMY, protoporphyrinogen/coproporphyrinogen III oxidase; HEMG, menaquinone-dependent protoporphyrinogen oxidase; CHLD, magnesium chelatase subunit D; CHLH, magnesium chelatase subunit H; CHLI, magnesium chelatase subunit I; CHLM, magnesium-protoporphyrin *O*-methyltransferase; CRD, magnesium-protoporphyrin IX monomethyl ester cyclase; POR, protochlorophyllide reductase; DVR, divinyl chlorophyllide a 8-vinyl-reductase; CHIG, chlorophyll/bacteriochlorophyll a synthase; CAO, chlorophyllide a oxygenase; PSY, phytoene synthase; PDS, phytoene desaturase; ZISO, zeta-carotene isomerase; ZDS, zeta-carotene desaturase; CRTISO, prolycopene isomerase; LCYE, lycopene ε-cyclase; LCYB, lycopene β-cyclase; ZEP, zeaxanthin epoxidase; VDE, violaxanthin de-epoxidase. Heatmaps were constructed based on the values of $log_2$RPKM.

Fig. S5

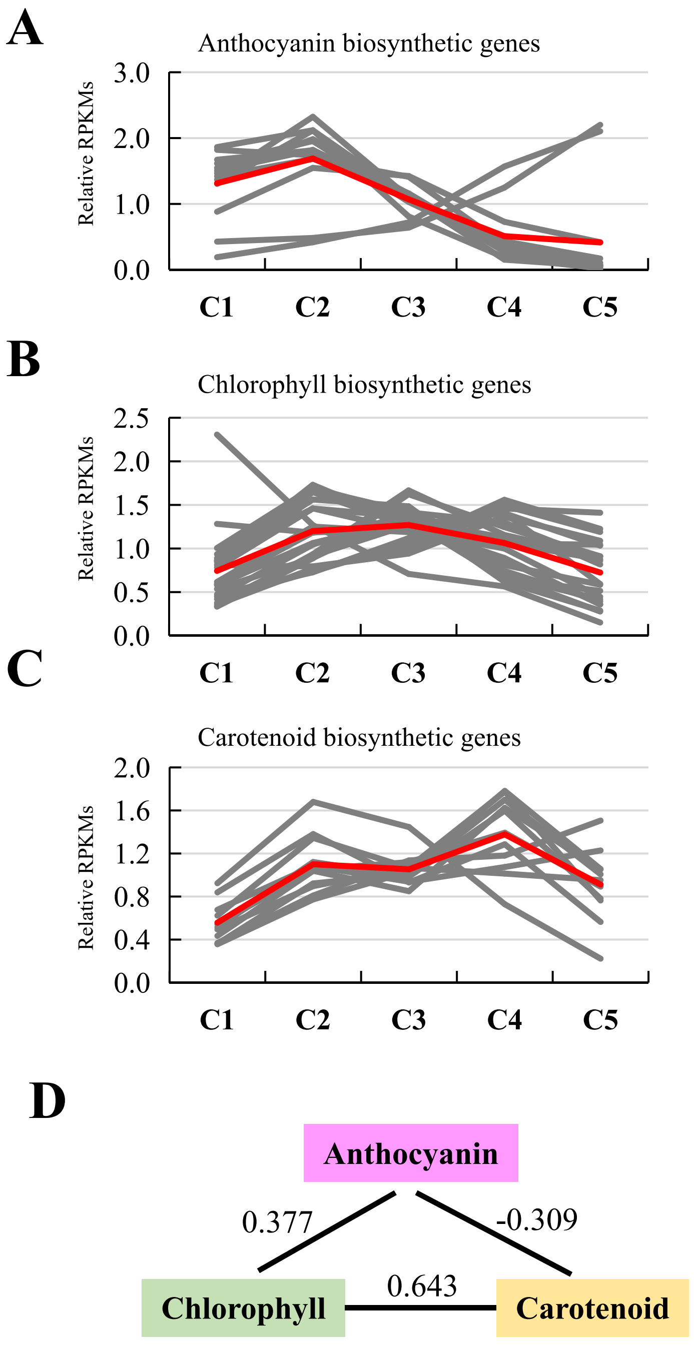


Fig. S5. Relative expression trends of the structural genes for the three pigment biosynthetic pathways. (A-C) The relative RPKMs for anthocyanin, chlorophyll, and carotenoid biosynthetic genes. Relative RPKMs for each gene were calcultated from RPKM/mean RPKM of the five stages. Each of the grey lines represent the expression trend of one structural gene. The red lines indcate the average trends of the pathway genes combined.

(D) The Pearson's coefficients among the three pathways calculated from the average trends shown in red of (A-C).

# Fig. S6

## A

***pro-IpCHSD-337***

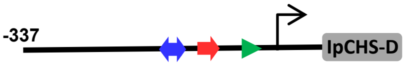


TAATATCTATACCACAAAATACCCAGTCTATTTTAAAGTTATTTTCCAACACGGGATACTTATTCAGACAATCAAGTCAT
GCATAACAATTATGAATTGCCGATTGTGGACGGTTTGCTGAGCCGGGTGAGG**CACGTG**ATTCCT**AGCTACC**ATATAAGTC
CATTATAACGTACACTTTAAAATCC**TATATAA**ATACCATGCCCTATACCCTATGACAAACAGAACATACAATTGCAGTTA
GTTAACGCCGCTTGTCTGTTAGTAACTACATTTAACGTACATCCGTTACTCTGCAATACCCTCAATTCTCCCACCCAAAC
AAAACGCCGGCGAAGAA**ATG**

***pro-IbCHSD-555***

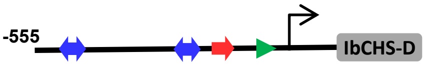


AGGTAC**CACGTG**AATGATTACAAGTTTGATTATTATCAAAATCCTCATGGTCCAGCCTGTCACACATGATCTTCCTGATG
TGATTAATTTACAATCTATTACACAAGGACAATGTTCATTCGGCCTTTTATTGTGAATATCAAAACCATTTATTTGTACG
TTGTTGAAGAATTCTTATTTAGGCATACTTCAATTCATACCATCCATAGTCCATACATAGTGTTTTTAATAAAAATTGTA
TCTCTCTTTAATTTGCAAAGATTAATTATTTTCACTTTATTGAGATGAGACAAAAGTCATAATAACAATTTTTAATTACC
GATTGTGGAAGGTTTGCTGATCAGCCGGGTGAGG**CACGTG**ATTCCT**AGCTACC**ATCCATAACTCCCATAAGTCCATAATA
ACACTTTAAAATTTG**TATATAA**ATACCATGCCCTACATACACCTTATGCCAAACAGAACAATTGCAGTTATTAACGCCGC
TTAGCTTGTCTGTTAATAACTACTCTACATTTAACGGAATAGGCACCCAAACAAAACGGAAAGAAGAAGGATCCG**ATG**

## B

***pro-IpDFRB-191***

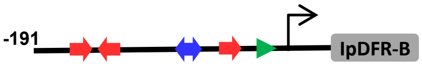


CGTC**ACCAAAC**GGGG**GGTTGAT**ATCGAGTATGAAGCACG**CACGTG**CATCGACGAGCGG**ACCAAAC**AGTCATGAGCTTAAC
TAATAACGTG**TATAAAA**CTTTGATCATTCTATAAGGATTTTCACAATATAATTCAACAACCTTATACTTTAATTTCTTCG
GTAGAAGCTTAGCTTATTGATCCGAGGAAAA**ATG**

***pro-IbDFRB-308***

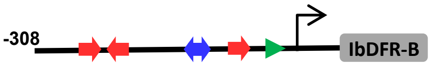


ATTAAGCGTTGAAGTGTGGTTAACCATGCTAACATGCGGTGTTTTAATAATAATATTATTATTATTGTTCCAATAATAGA
AAGGTTGTGTTGTTGGGTCAC**ACCAAAC**GGGG**GGTTGAT**ATCGAGTATAAAGCATGCACG**CACGTG**GTTGGCTTTTCAGT
CTATCTATATCGACGAGCGG**ACCAAAC**TGTCATGACCGTG**TATAAAT**CTTTGATCTTTCTATAAGGCTTTTCACTTGCAA
CATATCGACAATTCAACAACCTTAATTATACTTTAATTTCTTCGCTTATTGATCTGAGCAAAAATATA**ATG**

## C

***pro-IpbHLH2-348***

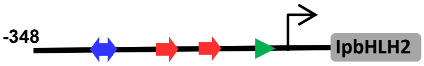


ACAGAACTCACCGATAGTAATATGATA**CACGTG**GGACACATAAAAGGCAAGGACAAAAACCCAATCTTGAATTCCCCTC
TTTTGTCGTGTCTTTCCATAGTCCCTATACCCGACCGGTTGAC**ACCAACC**AGTCAAGATCCAACGTAACTCCCCGACAA
ATAAATTCGATTC**ACCAAAC**CTTAACCCTCTCATCTACTTCACTGCCCTCTCCTTCAACGTAACTCTTTCA**TATGTAAT**
CCACTCTGTCTAGATCTTCCATCTTCAATTCATCATCATCGTCATCGAAATTCCACCGGAGATCCGATCCGCCATGCAT
GCATCACCTCCTCCTCCGCTTCGTCTATAGTA**ATG**

***pro-IbbHLH2-892***

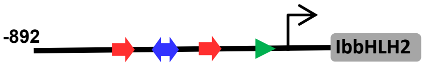


AGGTACCTGGTCTGCGAGTTGTTACAGATTAATAAAATTTATTCAAATAAACTCTCAGATAATAAAATGGGGTTTCTTA
TAAAAAAAAATAAAAATTAAAAATAAACTATAGGAACAGAAAATAGTCATACTCTTGATAAATAAGAGATGATGAAGAA
TTGAAGACCTCTTGTGCGCAGAGTGCACAGGTAAGCGATGAAAACTTGAAGCCAGATAAGGCAACTTACAATTTACCTC
TACTAGAAATTCTTAGGTAGTATGTGGTTCTAAACGTTTAGAATTAATTAGAGTTGTCGCATGGCAGCTTGATCAACTG
GTCACATGTGTGAAGTTTAGAAGAAGAAATCAGGGATCGAGTCTCATCAGTGGTAATGTAGGAGCAACCCCTTAAAGTG
AGGGGGTCCTTGTGTCCGGTTTAGTCCACTGAGGCTCAAATCCACCCCCATATGAGGGTGAAATCGGGTGCCACTAAAT
CACAAGTCTTTGACAGAAGAATTAAATAGAGCTGAAAA**ACCTAAC**TAAATTTATAATAAACAAAATAAAAAAAATAAAA
AGTGACACTAATATAGTAATATGATA**CACGTG**GGACACATAAAAGGCAAGGACAAAAACCTAATCTTGAATTCTCCTAT
TTTGTCGTCTCTTTCCCCAGTCCCTATACCCGACCGGTTGAC**ACCAACC**AGTCAAAATCCAACTCCCCGACAAACAAAT
AAATTCAACCTTAACCCCTTCAACTCTTAATTTCA**TATGTAAA**CCACTCTGTCTCGGATCTTTCCATCTTCAATTCATC
ATCGTCACGCAAATCTGTGTTGCAGATCAGAGATTCCACCCCCGCCATGCATGCATCGCCGCCTTAATTAGCTTCTCTG
TAATCTATATATAGTAGGATCCG**ATG**

# Fig. S6 continued

## D

***pro-IbCHSE-1069***

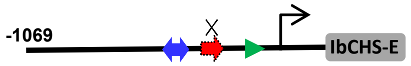


AGGTACCAATTCACGGCTGTAGCAAACTCCTCTATTTCTATTAATTTGTGGTTTCATGGCAAAACCGCACTATATTCAAT
ATATGAACCACACTTCTTTTAAATTTCAATTTTCAATTTACATGAGAAATTGGAATAATGGATTATACCATAATATTATT
GGGAAATTGGGAAATGACTAATGAGGTAATTGGGAAATCAGAAATGTAATTAGAAAATTAGGTAAGAAAAGGATTATTGT
AGCTTTCTCAAAAAAAAAAAAAAAAGGATTATTGTAGCGTGGGAAATTTGGCAGATGAGAGTAAATAACCAAAGTAACAGT
GGAAGT**CACGTG**CCGCCC**AGCTgCC**TGTCCTTTGGATTTACGGG**TATAAAT**ACCACTTAGCTTCTGCACATTCTACGCAC
TCTGAGTCTCTCTGATCTTCAAACAATTAAGTAATTACATTATATTTTGTTCTCTGGTCGGCCCGTGGCATCATATTCCT
ACCTGCTAGAAAATGGTGAGCGTCGAGGAGGTACGGAAGGCGCAACGTGCCCAGGGTCCGGCCACCATCATGGCTATAGG
AACCTCCACTCCCCCTAATTGCGTTGATCAAAGCACCTATCCAGATTATTTTTTCGTATCACCAATAGCGACCACATGG
TTGAACTTAAAGAAAAATTCAAACGCATGTGTACGTAAAATTACATGCTTTATTTTTCCCTTTCCTAGCTTCTCCATCTC
TTCTTCTCTTTTATTAGAACATGTCACTTTAATTTTCTTGTTAATTATGAATGTGTATATATAGATGTATATTTCTTTTT
CTAGTCAATTAGGATAACGATACAATCTAAAAGAAACAAGTTATGACAGCAATCCCAACTATAATATTGGTTGACTTGAT
AACTATAAGGTTACAAATTTAGTCCCAATGGGAACTGCCTATTGTTAGACCTTGCATGTGGTCCTTTGCTGCTAGGATCA
TAGAGTAGGGTTTATTCTGTGCATACTCTTGAGTAGTAGTAATGGCTGATGTTTATAGTTTTATACCAAATTGGGTAATG
TTTACTATATACATGCAGGTGAAAAATCA**ATG**

## E

***pro-IbMYB4c-805***

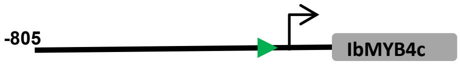


AGGTACCGGTGATCGAAGGCATGGAGATCATCTTGATAATTTCATAGTTTGTAACTATTCAACCTCCATATATAAAGCAT
TCATCATCTCTAATATGTGCATATATATTATCATTTTTCATAAATAAAAAATGGAAATATTACAGGTTTTTTGTTAGATC
ACGAGATGACTTGAGTATGTCCTAAATATATGGAACATTTTTAAGCCCTTATTATATTTAGACTAATTCTTGTTCGTACC
AAGCTGATATAATTATAGTGTAAAATGTTGACCATATTGATTTTTCTATATCAGAGGGGATTTTGAAGTCAGAGAAGTAT
TACAGGTTTTGCATATGTTTATACAAACAATACATACATATATAGTAGATTGTGTGCAGTCATGCTGGGCGAATTACACA
CAATTTAGTGTGTGCTTTTGTGTAGTAATATAATTTATGTGCACTTAAAAGAGTTTCATCAGAAGGAGTTGTCTCATTTG
ATTATATATATATATAGAATCACGATCAGGTGCAGTCGTGCTCTCCCGTGCGGCCGTGCGGTTCACACTACTCAATGTTAGG
AAATACACCACTACTCAATGTTAAGAAACACACCACACAAATATGCACGGTGGTGTGTGACCGCACGGCCGCACTTGAAA
T**TATATATA**CCCAGTCGTCCAATCAAGCTAAGGAGTGAATACACACACATGACACACCCAAGGCTCCTTCCTACCTGTCT
CCCTTAAAAATACTACTCTCTCTCTCTCCCTCTCTCTCTAAAAAAAAAAGGGAATAATTTGAAAAGGAGAAGAGAAGG
ATCCG**ATG**

## F

***pro-IbMYB27-768***

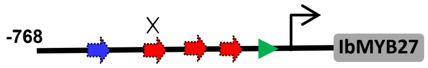


AGGTACCAACATCGGTCCCAATCCAAAGCTCATCGTATTATATACATAGCGCCTCGCTGTCCCAAGTTTATCAGTACGT
AAGCTACACTGAGTTGATCATTTATACTTTATAACAAAACTAAATGGTGCTTATTTGCCAAGGACCTTGTGATCCAGTG
ACATCAAACCCTTCCCTTTATATGGGAGGTGTCTACTCACTATGCACAAAAATCCCTCTCATTTTGAGAGGTTATTCTC
A**CACGTA**GGTGTCTATTGTGACAAATTTCATTTTTCATCTGTGTGATCAATTGAGTTATGCATGTAAGTATTGGGTGCA
ACTCTTACACTACGACTGCTAGAGTTTGATTATGTATCATTACTATAATTAACCTACTTAAAAATCAACTG**AGCTgCC**T
GTGCGATAATAATTGCTCGTGCATTATAGTTGAGGTAGTACCATAGAAAGAAAAATTGTATTGATTATGAATTTTATAA
AAGTGGGTGGTGTGAGTAAGGCATGCATTGCATACATACTAGAGCATGCAGGGCCAAACCACCGGTCAACTCTCTCATG
GTCCTCCTTTTATTACATAAAACACTATAAAGTGTAA**ACCACCC**CCACACCACACCTC**ACCCACC**ACCGCCTCCGTCTT
CACGCAACTA**TATATAA**TTACGCTCTCATCATATATTTTCTATATATATACAACATAGCCAAGGAGTGTACTTTTCTTC
CCGGATCCACACCCCTAATTCACTTCTTGTCTCTCCGACAACTAACGACTGTCGACT**ATG**

Fig. S6. Potential *cis* elements of the MREs and BREs on the promoters for anthocyanin MBW complexes. (A-C) The sequences of cloned proximal promoters for *IbCHS-D*, *IbDFR-B,* and *IbbHLH2* were shown with their homologous promoters of *I. purpurea* as reported by Zhu et al. (2015), which were verified with detailed experiments.

(D-F) The sequences of cloned proximal promoters of *IbCHS-E*, *IbMYB27* and *IbMYB4c*.

Proximal promoter architectures of the promoters were shown. MREs and BREs are indicated with red and blue arrows, respectively. The TATA boxes are indicated with green triangles. The sites are marked for putative MREs (in red and bold), BREs (in blue and bold) along with the expected TATA boxes (in green and bold). Mutated MREs are indicated by the crosses. The 5'utr region was underlined with the starting codon ATG emphasized at the end.

# Fig. S7

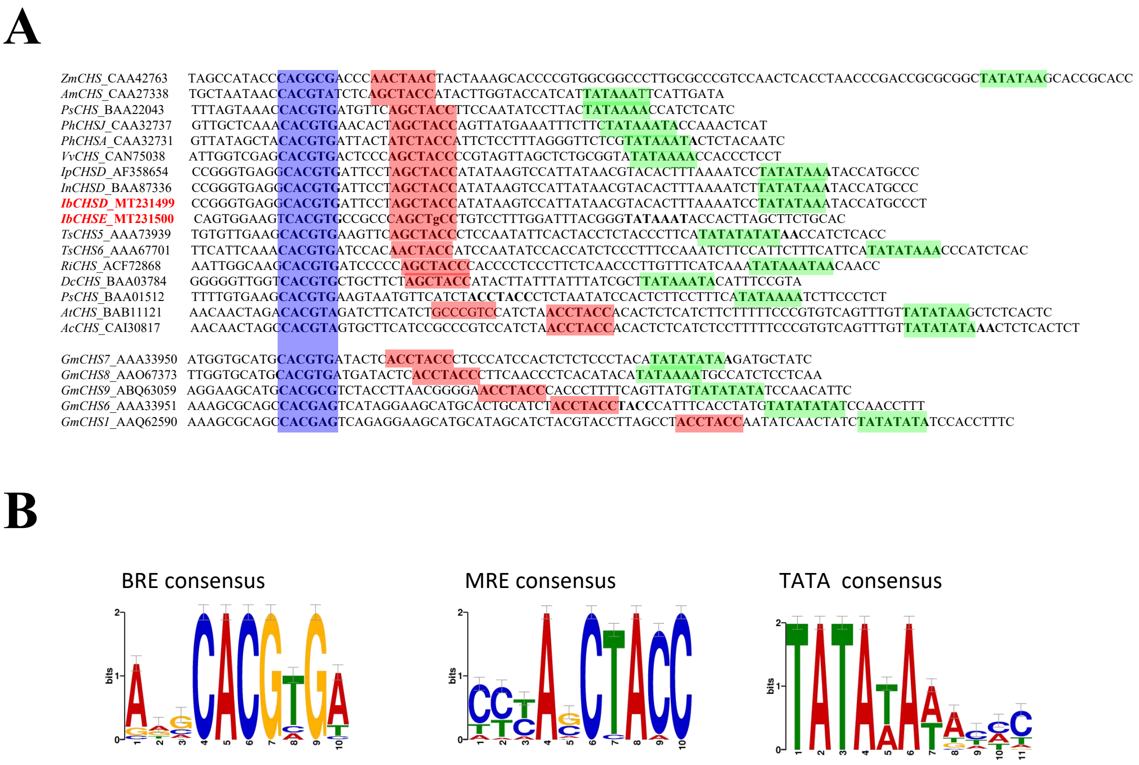


Fig. S7. Conservation of the *cis* elements for the MBW complex on *CHS* promoters from multiple species.

(A) The *CHS* proximal promoter fragments from various species with MREs (in red and bold), BREs (in blue and bold), and TATA boxes (in green and bold) marked. The promoters for *IbCHS-D* and *IbCHS-E* were in red and bold. *Zm* for *Zea mays* (maize), *Am* for *Antirrhinum majus* (snapdragon), *Ps* for *Pisum sativum*, *Ph* for *Petunia hybrida*, *Vv* for *Vitis vinifera* (grape), *Ip* for *Ipomoea purpurea*, *In* for *Ipomoea nil*, *Ib* for *Ipomoea batatas*, *Ts* for *Trifolium subterraneum*, *Ri* for *Rubus idaeus*, *Dc* for *Daucus carota*, *At* for *Arabidopsis thaliana*, *Ac* for *Arabidopsis croatica*, *Gm* for *Glycine max*.

(B) The consensus sequences of BREs, MREs, and TATA boxes for the above *CHS* promoters concluded by MEME (http://meme.nbcr.net/meme/).
